# Dynamical Mechanism Underlying Scale-Free Network Reorganization in Low Acetylcholine States Corresponding to Slow Wave Sleep

**DOI:** 10.3389/fnetp.2021.759131

**Published:** 2021-10-25

**Authors:** Paulina Czarnecki, Jack Lin, Sara J. Aton, Michal Zochowski

**Affiliations:** 1 Department of Mathematics, University of Michigan, Ann Arbor, MI, United States,; 2 Neuroscience Graduate Program, University of Michigan, Ann Arbor, MI, United States,; 3 Department of Molecular, Cellular and Developmental Biology, University of Michigan, Ann Arbor, MI, United States,; 4 Department of Physics and Biophysics Program, University of Michigan, Ann Arbor, MI, United States

**Keywords:** neuron model, networks, scale-free, acetylcholine, sleep, STDP, synchrony

## Abstract

Sleep is indispensable for most animals’ cognitive functions, and is hypothesized to be a major factor in memory consolidation. Although we do not fully understand the mechanisms of network reorganisation driving memory consolidation, available data suggests that sleep-associated neurochemical changes may be important for such processes. In particular, global acetylcholine levels change across the sleep/wake cycle, with high cholinergic tone during wake and REM sleep and low cholinergic tone during slow wave sleep. Furthermore, experimental perturbation of cholinergic tone has been shown to impact memory storage. Through *in silico* modeling of neuronal networks, we show how spiking dynamics change in highly heterogenous networks under varying levels of cholinergic tone, with neuronal networks under high cholinergic modulation firing asynchronously and at high frequencies, while those under low cholinergic modulation exhibit synchronous patterns of activity. We further examined the network’s dynamics and its reorganization mediated via changing levels of acetylcholine within the context of different scale-free topologies, comparing network activity within the hub cells, a small group of neurons having high degree connectivity, and with the rest of the network. We show a dramatic, state-dependent change in information flow throughout the network, with highly active hub cells integrating information in a high-acetylcholine state, and transferring it to rest of the network in a low-acetylcholine state. This result is experimentally corroborated by frequency-dependent frequency changes observed *in vivo* experiments. Together, these findings provide insight into how new neurons are recruited into memory traces during sleep, a mechanism which may underlie system memory consolidation.

## 1 INTRODUCTION

Sleep is crucial for normal cognitive functions (Deak and Stickgold, 2010; Wilckens et al., 2014), however, the mechanistic underpinnings of its brain-specific functions are largely unknown. Studies have implicated the role of sleep in mediating the overall homeostatic depotentiation of brain connectivity after active storage during waking (i.e. synaptic renormalization hypothesis (Tononi and Cirelli, 2014)) as well as its participation in memory storage and consolidation (Benington and Frank, 2003; Stickgold, 2005).

Numerous results have shown that sleep promotes changes in network organization following the learning experience in order to consolidate new memories throughout the brain (Maquet, 2001; Diekelmann and Born, 2010; Ognjanovski et al., 2014; Durkin et al., 2017; Ognjanovski et al., 2017; Klinzing et al., 2019; Puentes-Mestril et al., 2019). For example, in hippocampus, cellular indicators of synaptic strengthening increase during sleep in the hours following spatial or contextual learning (Ribeiro et al., 1999; Ognjanovski et al., 2014, 2017; Durkin et al., 2017). Other examples include motor cortex of adult mice, where dendritic spine growth occurs in a sleep-dependent manner immediately following motor learning (Yang et al., 2014) or visual cortex of juvenile cats and adult mice, where indicators of synaptic strengthening are present during sleep after a novel visual experience (Aton et al., 2009, 2013; Puentes-Mestril et al., 2019). In contrast, sleep deprivation was associated with the disruption of the strengthening of glutamatergic synapses, and the reduction of dendritic spines in CA1 and DG pyramidal neurons of hippocampus (Havekes et al., 2016; Raven et al., 2019).

The brain’s neuromodulatory milieu changes significantly between different vigilance states (i.e. wake, rapid eye movement (REM) and slow-wave sleep (SWS)) (Watson et al., 2010; Lee and Dan, 2012). One of these neuromodulators, acetylcholine (ACh), can have dramatic effects on neuronal properties and synaptic transmission (Watson et al., 2010). Throughout the brain, wake and REM states are associated with high levels of ACh, while slow wave sleep (SWS) is characterized by low levels of ACh (Watson et al., 2010; Lee and Dan, 2012). This leads to the question of how these different vigilance states, and thus neuromodulatory milieu associated with them, affect memory consolidation (Genzel et al., 2014).

On the brain systems level, it has been postulated early on that ACh may regulate information flow between the hippocampus and neocortex. According to this hypothesis, neocortical signaling to the hippocampus is predominant during wake and REM, while in NREM, the information flow may be reversed, with memory traces stored in hippocampus being transmitted back to neocortex (Buzsaki, 1996; Buzsaki, 1998; Hasselmo, 1999; Gais and Born, 2004; Power, 2004; Diekelmann and Born, 2010; Klinzing et al., 2019). This hypothesis was supported by experimental findings showing increased correlation between neocortical and hippocampal activity during SWS (Sirota et al., 2003). Other studies directly correlated the increase of post sleep performance with ACh release. Specifically, Gais and Born (Gais and Born, 2004) shown that experimental subjects experienced improvements in declarative and procedural based memory tasks following sleep. Subsequently, they showed that by increasing the bioavailability of ACh through the application of acetylcholine esterase inhibitor (physostigmine), effectively reversed the declarative memory task improvement observed after sleep.

**FIGURE 1 |:**
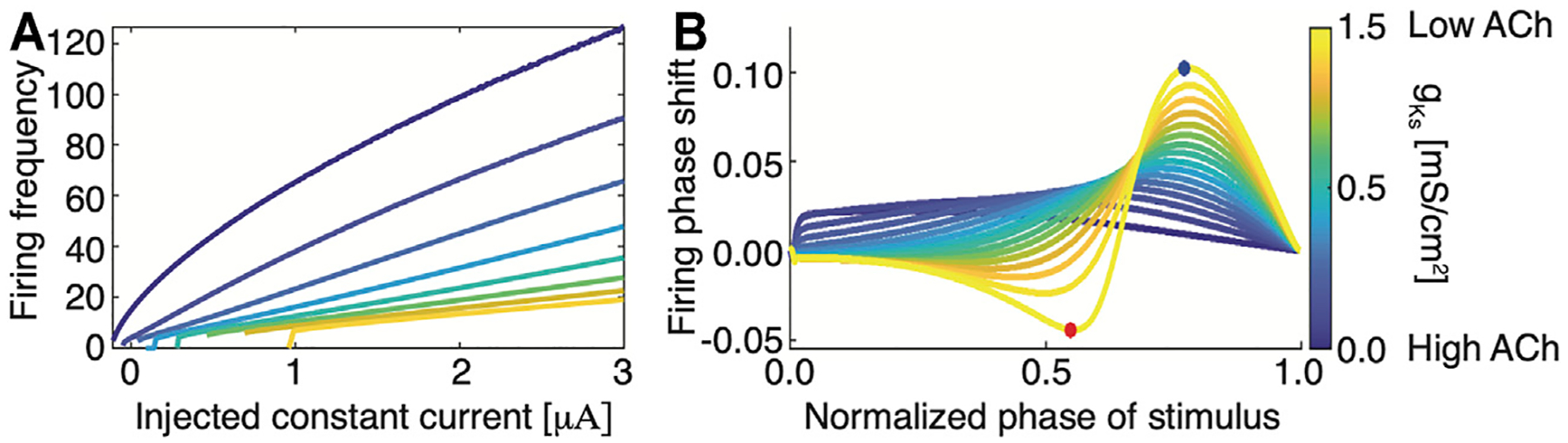
Transition from Type I to Type II membrane excitability as a function of magnitude of m-current conductance, gKs, regulated by changing ACh levels **(A)** Neuronal Input-Frequency (I-F) curve for different values of *g*_*Ks*_ (blue, *g*_*Ks*_ = 0 *mS*/*cm*^2^; yellow, *g*_*Ks*_ = 1.5 *mS*/*cm*^2^). **(B)** Phase response curves (PRCs) for different values of *g*_*Ks*_ (blue, *g*_*Ks*_ = 0 *mS*/*cm*^2^; yellow, *g*_*Ks*_ = 1.5 *mS*/*cm*^2^). Blue and red dots denote maximal and minimal phase shifts, respectively. The PRC is measured by comparing perturbed vs. unperturbed firing periods when neurons fire at a fixed frequency. Type I neurons have a strictly positive PRC (blue) while Type II neurons have a biphasic PRC.

On the other hand, at the cellular level, changes in the level of ACh affect neuronal properties (via muscarinic (Haga, 2013; Kruse et al., 2014) and nicotinic (Albuquerque et al., 2009) receptors) including neuronal excitability, firing responses to input current, and the neuron’s phase response curves (PRCs)—the change in spike timing in response to perturbation (Fink et al., 2013; Ermentrout, 1996). In particular, muscarinic receptors facilitate cholinergic regulation of outward M-type potassium current (Stiefel et al., 2009). This outward current reduces neuronal excitability and results in a Type II PRC. High concentrations of ACh blocks these receptors, resulting in a Type I excitability during wake and a Type II excitability during SWS. Phenomenologically, this transition in spike generation is described through a saddle node limit cycle bifurcation in the case of a Type I excitability and a Hopf bifurcation in the case of a Type II excitability (Börgers, 2017; Stiefel et al., 2008). Type I excitability is generally characterized by a steep input current-frequency (I-F) curve with continuous approach to 0 Hz frequency as a function of external input (Figure 1A; blue curve). At the same time, the PRCs takes on only positive values meaning that brief excitatory input is only capable of advancing phase firing of a neuron (Figure 1B; blue curve). In contrast, Type II excitability in characterized by relatively flat I-F curves with discontinuous jump in firing frequency at the minimal firing frequency (Figure 1A; yellow curve). Furthermore, the PRC changes from a negative phase shift response for lower phase values to a positive phase shift response for higher phase values (Figure 1B; yellow curve). This transition from Type I to Type II excitability has been shown to increase synchronizability in the network (Ermentrout, 1996; Börgers, 2017).

Thus, when ACh levels are high in the brain (e.g. in wake), neurons will exhibit Type I behaviors: they spike in response to an incoming stimulus, with spiking frequency generally proportional to the magnitude of the input (Stiefel et al., 2009; Fink et al., 2013). Here, the I-F curve is steep, indicating that increasing input leads to greater spike frequency responses. Additionally, the neurons exhibit a Type I PRC, which reduces the capacity for the synchronization of firing between neurons. During SWS (low ACh), the I-F curve becomes flat, reducing neuronal frequency response to incoming input. This has the effect of homogenizing firing frequencies in the network (observed also experimentally (Miyawaki et al., 2019)) while aligning neuronal spike timings more readily to phasic inputs–i.e., a Type II PRC (Stiefel et al., 2009; Fink et al., 2013). This transition from Type I phase response to Type II facilitates the increased synchrony associated with slow-wave sleep (Babloyantz et al., 1985; Ermentrout, 1996; Fink et al., 2013).

We have previously investigated the reorganization in network dynamics of neurons with homogenous connectivity density under globally uniform ACh mediated excitability changes (Fink et al., 2013; Roach et al., 2018). Separately, we have also investigated network dynamics when the neurons in the network are differentially modulated by spatially heterogenous ACh levels (Yang et al., 2021). Here we investigate how changing levels of ACh, which modulates neuronal excitability via muscarinic receptors, can globally mediate changes in the information flow in networks having heterogenous connectivity, and subsequently initiate structural reorganization within the network. We specifically study how changes in ACh mediated neuronal excitability, coupled with network level spike timing dependent plasticity (STDP), mediate scale-free network reorganization.

The large-scale network activity of the brain is consistent with behaviors expected from highly heterogeneous connectivity. For example, lognormal firing frequency distributions are found in brain structures including the neocortex, hippocampus, cerebellum, striatum, and the midbrain nuclei which may indicate scale-free network structures within these regions (Mizuseki and Miyawaki, 2017; Scheler, 2017; Clawson et al., 2018). In concordance, human brain imaging has revealed functionally scale-free networks across the brain (Eguiluz et al., 2005). These scale-free networks are characterized by a connectivity distribution where a few elements (e.g., neurons) have a very high number of synaptic connections, while most have very few connections. These connections, on the meso-scale, are rarely bi-directional. Often, the highly-connected hubs can have primarily incoming or outgoing connections (Hillebrand et al., 2016; Budak and Zochowski, 2019).

Our results indicate a dramatic, state dependent change of information flow throughout the network, wherein hub cells first integrate information in a high-ACh state, and then transfer it to the rest of the network in a low-ACh state. We find that this effect is surprisingly robust against various levels of noise and network inhibition, and therefore may be prevalent throughout various brain modalities affected by ACh changes. We hypothesize that this switch provides a dynamical mechanism by which neurons are recruited into memory traces during sleep to promote systems memory consolidation, within and across brain modalities.

## 2 RESULTS

### 2.1 Cholinergic Modulation of Network Dynamics

To evaluate the effects of state-dependent ACh modulation on network dynamics, we performed simulations of excitatory networks composed of 250 biophysical models of neurons that included an ACh-regulated, M-type potassium current. Since the potassium current is inhibited by ACh, we modeled cholinergic modulation by varying the maximal conductance of the M-type potassium channel (*g*_*Ks*_), which is inversely proportional to the relative level of ACh release (see Section 4). To implement a highly heterogeneous network structure, we used scale-free connectivity schemes (Barabasi, 2009), which gave rise to a small subset of neurons with large numbers of incoming/outgoing connections (hubs) while the rest of the population had few such connections.

Given the non-uniformity of nodal degree across a scale-free network, we wanted to assess the effects of different connection schemes on global dynamics. By applying the probabilistic connection flipping algorithm (see Section 4), we were able to establish five scale-free networks with different distributions of in-degree percentages. By setting the probabilistic in-degree value *p*_*in*_ to 0.1, we created a scale-free network in which most of the connections within the hub neurons were directed outwards (Supplementary Figure S1). We refer to these networks as “strong hub outgoing.” Conversely, by setting *p*_*in*_ to 0.9, we obtained the reverse effect in which these connections are directed inwards (“strong hub incoming” networks). Since the total number of connections within the network was not modified, our algorithm resulted in an inverse change of in-degree percentage in the complementary neurons (non-hub neurons). We saw that the average in-degree percentage in the 25 neurons with the lowest degree did display a reversed connectivity pattern relative to the hub neurons. In addition, we created balanced networks (*p*_*in*_ = 0.5; Suppleemntary Figure S1), and two moderate hub incoming/outgoing structures, with *p*_*in*_ = 0.7 and *p*_*in*_ = 0.3, respectively.

**FIGURE 2 |:**
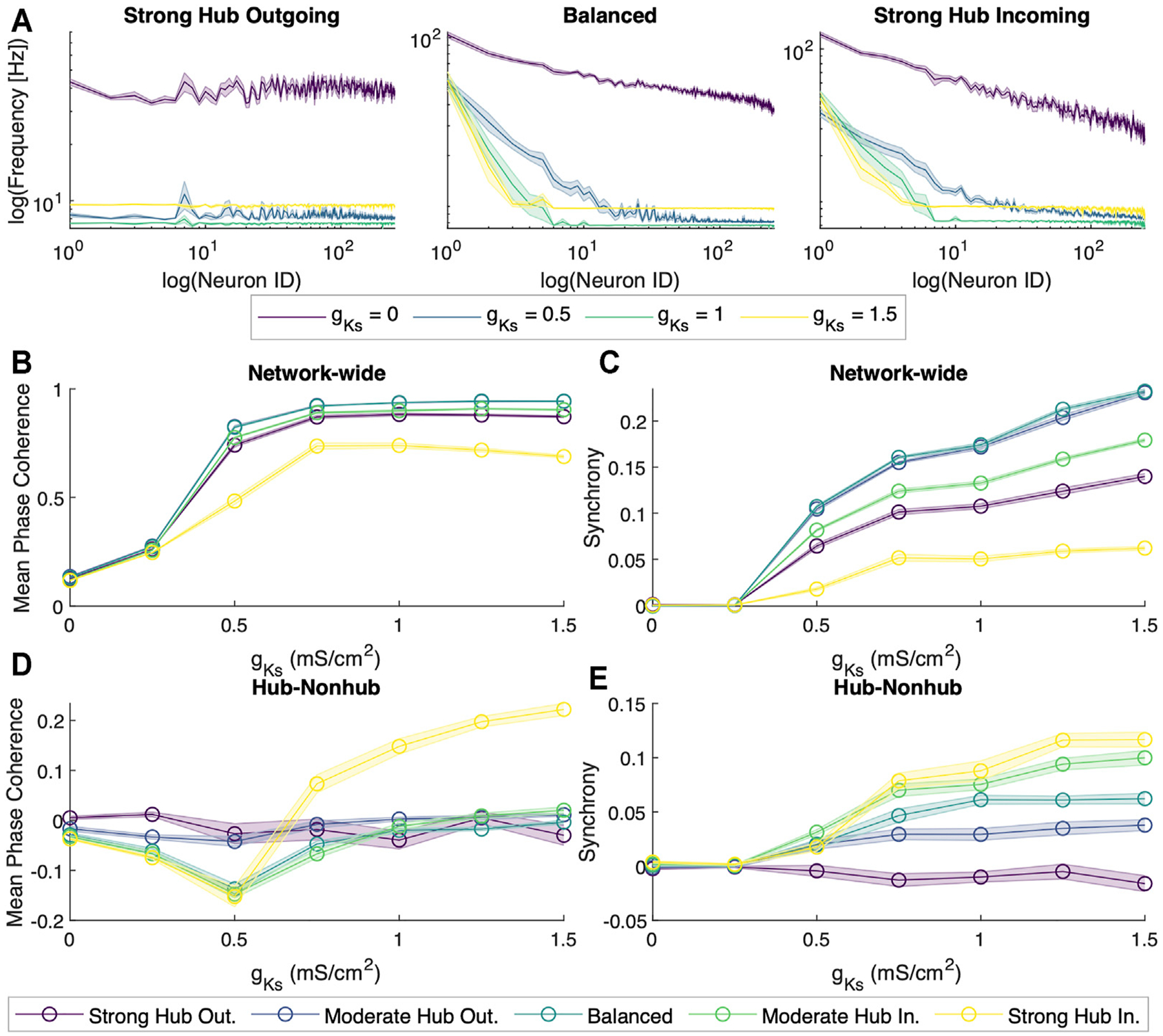
Dynamical properties of neuronal networks under different levels of ACh modulation. **(A)** Spiking frequency averaged across sets of 10 simulations for four values of M-current conductance (*g*_*Ks*_ {0, 0.5, 1, 1.5} mS/cm^2^, violet, blue, green and yellow, respectively) within the strong hub outgoing **(left)**, balanced **(center)** and strong hub incoming **(right)** network configurations. Shaded envelopes represent standard error of the mean. **(B)** Network-wide average of pairwise Mean Phase Coherence and **(C)** Cross Correlation were calculated across 10 independent simulations as a function of increasing *g*_*Ks*_ levels (x-axis) for five network configurations: strong hub outgoing (violet), moderate hub outgoing (dark blue), balanced (teal), moderate hub incoming green) and strong hub incoming (yellow). **(D)** Differences between hub and non-hub neurons for Mean Phase Coherence and **(E)** Synchrony as a function of increasing *g*_*Ks*_ for five network connectivity configurations, as listed above.

First, we interrogated network dynamics as a function of maximal conductance of the slow muscarinic potassium channel, *g*_*Ks*_. We observed that at *g*_*Ks*_ = 0 mS/cm^2^, neuron spiking activity seemed to be uncoordinated. However, as we increased *g*_*Ks*_, the neurons’ spiking began to synchronize (for sample rasterplots please see Supplementary Figure S2) and exhibit oscillatory activity with dominant frequency in theta band (for spectral analysis of reconstructed local field potentials (LFP) please see Supplementary Figure S3). The transition from the random firing pattern at *g*_*Ks*_ = 0 mS/cm^2^ to the increasingly synchronous firing pattern at *g*_*Ks*_ = 0.5 mS/cm^2^ was coupled with a drastic reduction in the overall spiking frequency (Figure 2A). Moreover, the spiking frequency homogenized as *g*_*Ks*_ increased, with non-hub cells firing largely at the same frequency while a few hub cells fired at significantly higher frequency for *g*_*Ks*_ ≥ 0.5 mS/cm^2^. In order to quantify the difference in the network dynamics across the different levels of *g*_*Ks*_, we applied metrics for spike coherence and synchrony by calculating the average Mean Phase Coherence (MPC, measuring the phase locking between neurons) and zero lag Cross Correlation (CC; measuring zero lag synchrony) (Figures 2B,C).

For all network configurations, MPC and CC increased as *g*_*Ks*_ increased (Figures 2B,C). However, the more balanced configurations (moderate hub outgoing and balanced) tended to have higher overall MPC and CC than the more extreme configurations (strong hub outgoing/incoming). Furthermore, we calculated the difference between average hub and non-hub MPC and CC scores (Figures 2D,E). For most network configurations, there were no noticeable differences in the hub and non-hub MPC, but the hubs were generally more synchronous than the non-hub at *g*_*Ks*_ levels higher than 0.25 mS/cm^2^. Additionally, we observed that for the balanced, moderate, and strong hub incoming configurations, the non-hub neurons had a higher MPC than the hub ones at lower ranges of *g*_*Ks*_.

**FIGURE 3 |:**
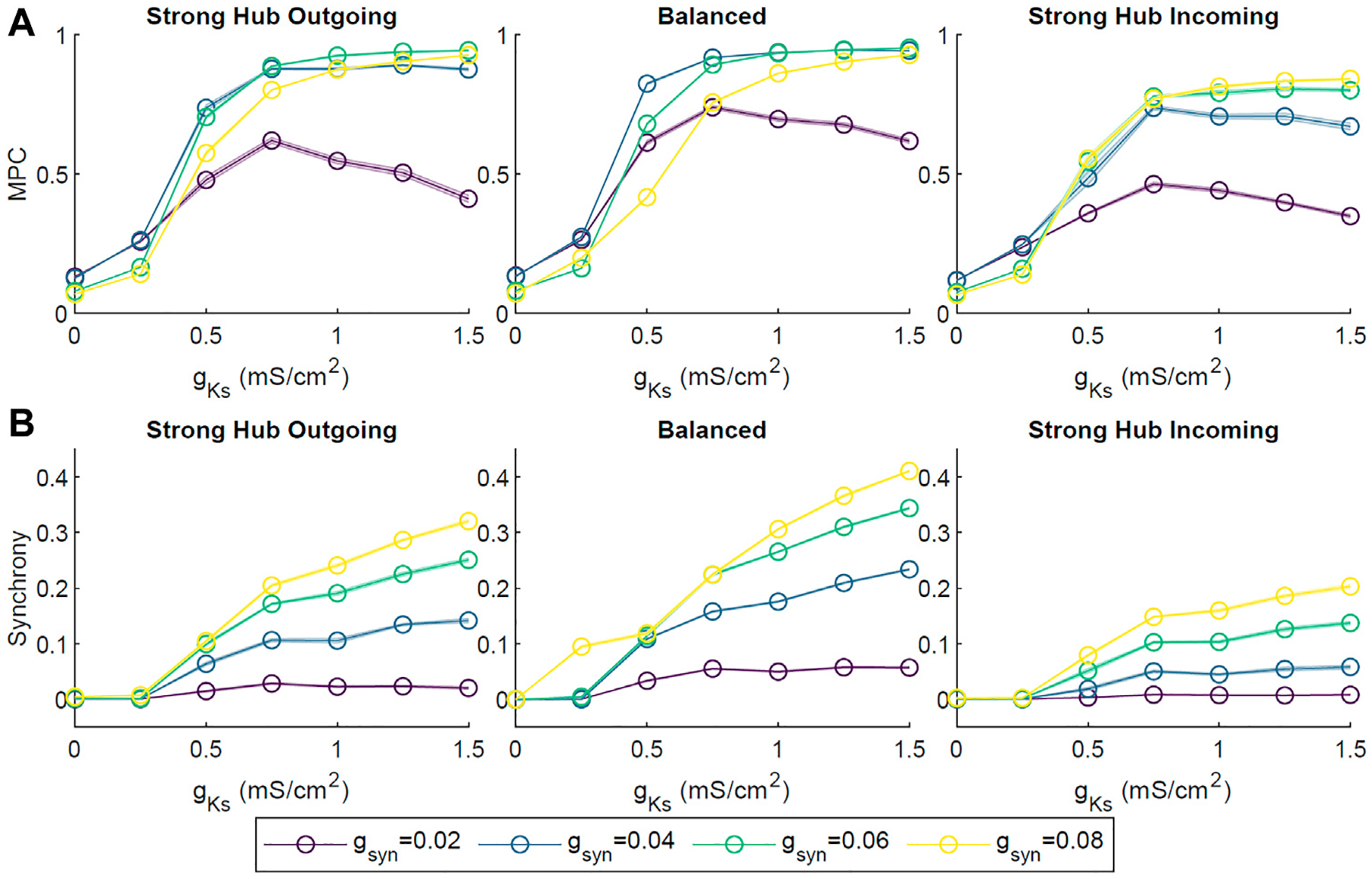
The effect of network connectivity strength on ACh-modulated network dynamics. Four network connectivity strengths were modeled by setting the synaptic conductance *g*_*syn*_ of every synapse to 0.02, 0.04, 0.06 or 0.08 mS/cm^2^. Simulations were repeated 10 times for each set of parameters across different *g*_*Ks*_ values and network configurations. Average pairwise MPC **(A)** and Synchrony **(B)** were evaluated, showing similar trends of increasing MPC and Synchrony with the increase in *g*_*Ks*_ across connectivity strength parameters.

**FIGURE 4 |:**
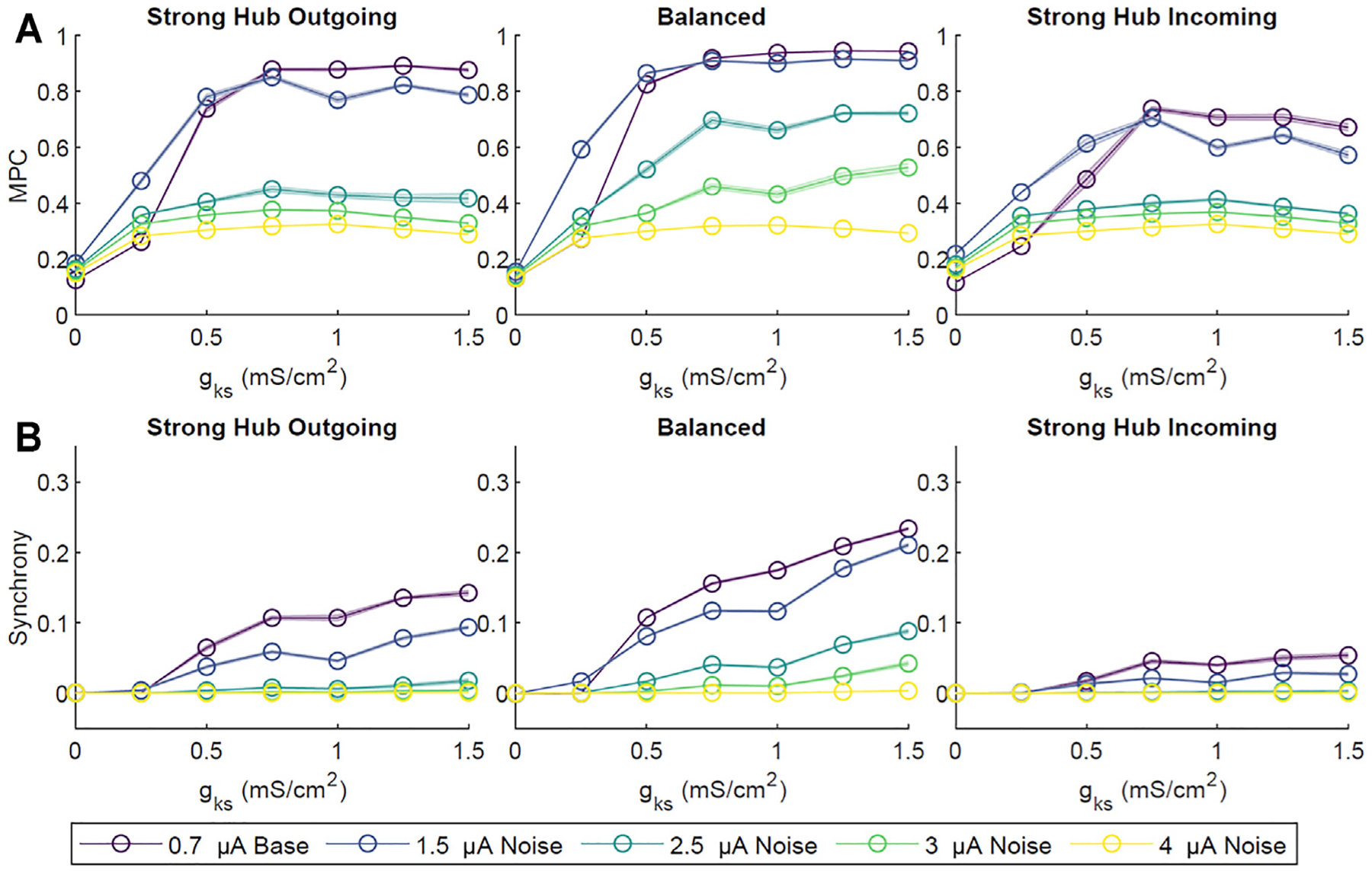
The effect of noise on ACh-modulated network dynamics. Four amplitudes (1.5, 2.5, 3.0, 4.0 *μ*A) of random, low probability (0.1% per time step) external direct current impulse paradigms were simulated and compared to our primary low amplitude (0.7 *μ*A), high probability (2%) current paradigm. Simulations were repeated ten times for each set of *g*_*Ks*_ value and network configuration. Network-wide, averaged pairwise MPC **(A)** and Synchrony **(B)** were subsequently calculated. Across all network configurations there was a general decrease in MPC and Synchrony with increasing noise amplitude. As with our primarily external current paradigm (0.7 *μ*A amplitude), MPC and CC increased with increasing *g*_*Ks*_. Shaded envelopes denoted standard error of the mean.

To assess the robustness of the phase coherence and synchrony measures for different connectivity frameworks, we investigated how these quantities changed as a function of *g*_*Ks*_ for different levels of connectivity strength (Figure 3) and external noise (Figure 4). Connectivity strength was represented by the synaptic conductance *g*_*syn*_ between two neurons and was initialized to the same value for all neurons. Here, we tested *g*_*syn*_ values from 0.02 to 0.08 mS/cm^2^. We observed similar behaviors across these connectivity strengths, with MPC and CC generally increasing as *g*_*Ks*_ increased (Figure 3). An exception to this was the weakest connectivity strength *g*_*syn*_ = 0.02 mS/cm^2^, which showed a reversal in MPC after peaking at *g*_*Ks*_ ≃ 0.75 mS/cm^2^. Interestingly, the balanced connectivity network obtained the highest measures of MPC and synchrony.

In these simulations, baseline external noise was defined as low amplitude (0.7 *μ*A), high probability (2% chance of initiating at a given time step, 200 Hz average), random current pulses. To investigate the robustness of network activity patterns against higher noise levels, we applied supra-threshold current between 1.5 and 4 *μ*A at a low initiation probability of 0.1% per time step. The overall network MPC and CC decreased with increasing noise amplitude (Figure 4), with the balanced network most resistant to high noise. In contrast, the strong hub incoming configuration was the least resistant, likely due to non-hub neurons receiving sparse inputs, thus having dynamics dominated by noise rather than network signaling.

### 2.2 Dynamics of E-I Networks

Since all results above were obtained from simulations of networks of only excitatory cells (E network), we investigated if the addition of inhibitory neurons (E-I network) would affect ACh-modulated network dynamics. To this end, we modified 10% of the neurons to have an inhibitory synaptic effect on the rest of the cells. While the excitatory neurons maintained their scale-free topology, connections to inhibitory neurons were uniformly random.

**FIGURE 5 |:**
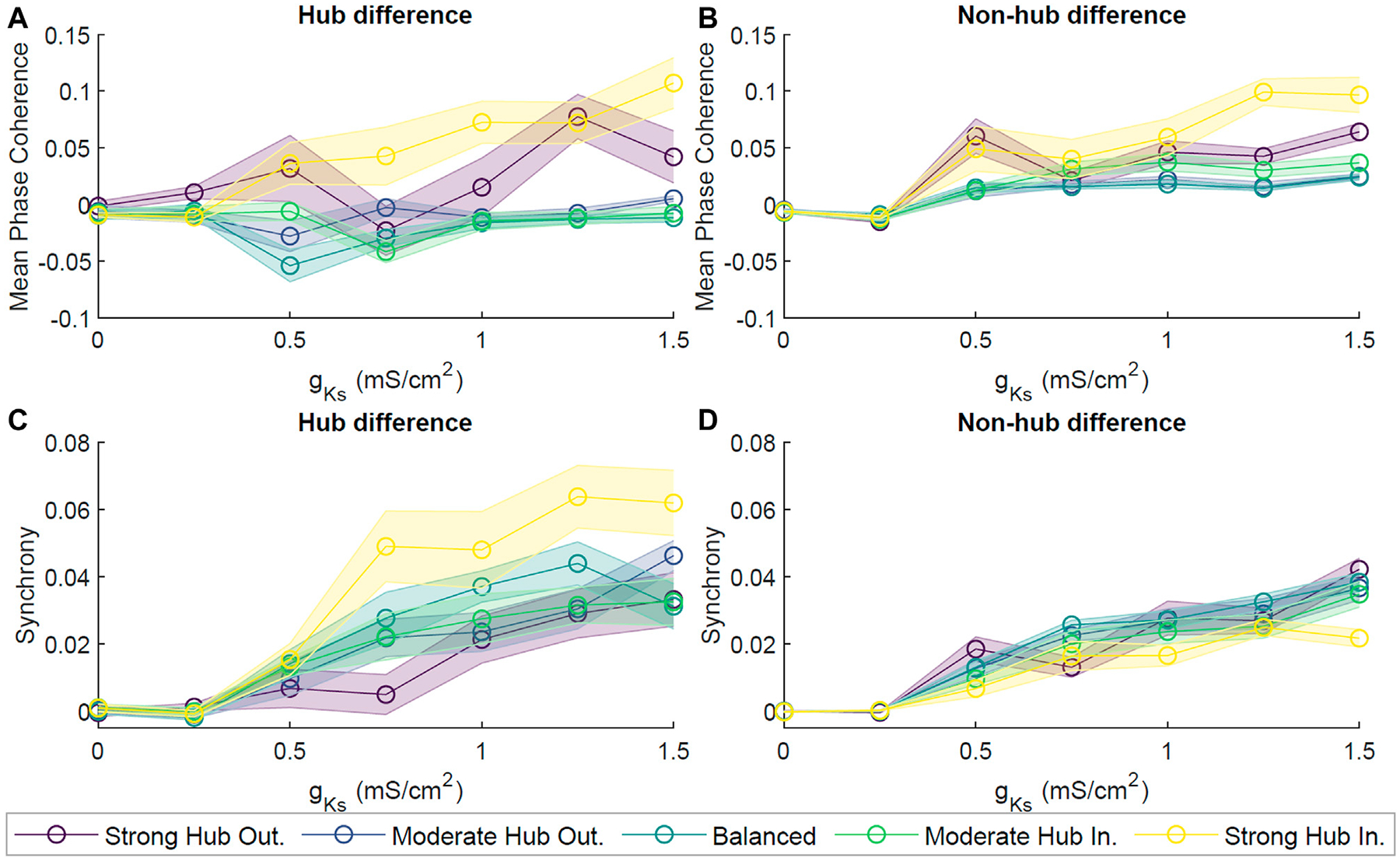
Dynamics of acetylcholine-modulated neuronal networks with excitatory and inhibitory neurons. E-I networks were implemented by allowing 10% of neurons to have inhibitory synaptic connections. Difference in Mean Phase Coherence between excitatory (E) networks and mixed (E-I) networks for excitatory hub neurons **(A)** and excitatory non-hub neurons **(B)**. Figures represent the mean across 10 simulations per parameter set. The difference in averaged Synchrony was calculated for hub **(C)** and non-hub **(D)** neurons. For both analyses, a positive score indicates a higher score of E networks than that of E-I networks, while a negative score represents the opposite relationship. The introduction of inhibitory neurons led to a general decrease in hub and non-hub synchrony as well as hub coherence.

As with the E network, we saw a trend of increasingly synchronous neuronal spike activity with increasing *g*_*Ks*_ (Supplementary Figure S4). This effect was quantified and validated with MPC and CC (figure not shown). To evaluate changes in coherence with the addition of inhibitory neurons, we computed the difference in the averaged MPC scores between that of all the excitatory neurons within the E-I network with that of the E network. We saw that for all network configurations, the non-hub neurons in the E-I network displayed lower MPC than those of the E network at *g*_*Ks*_ higher than 0.25 mS/cm^2^ (Figures 5A,B). On the other hand, the MPC for the hub neurons in the E and E-I networks were equal, with the exception of those in the strong hub incoming and outgoing configurations (Figures 5C,D). Changes in synchrony were assessed in a similar fashion, yielding lower synchrony in the E-I network for both the hub and non-hub groups.

To further investigate generality and robustness of this result, we compared coherence and synchrony within hub and non-hub cells for networks consisting of 10, 15, 20% of inhibitory cells (Supplementary Figure S5).

### 2.3 Temporal Organization Within Neural Firing Patterns

Next we investigated emergence of additional temporal ordering, between spiking patterns of neuronal pairs having different connectivity, in a network composed of excitatory only neurons. In addition to an increase in synchrony and coherence as *g*_*Ks*_ increased, we observed emergence of temporal asymmetry in the neuronal spiking activity (Supplementary Figure S2). Namely, the spiking of neurons with lower degree lagged behind neurons with higher degrees. To quantify this, we used a measure known as unidirectional average mean distance (AMD) between every pair of neuron spike trains (please see Section 4 and also (Wu et al., 2018)). A statistical AMD Z-score (AMDZ) less than −2 denoted a significant temporal locking between a pair of neuron spike trains *S*_*i*_ and *S*_*j*_, where *S*_*i*_ preceded *S*_*j*_. Conversely, an AMDZ value greater than 2 denoted a significant temporal locking in the opposite direction.

**FIGURE 6 |:**
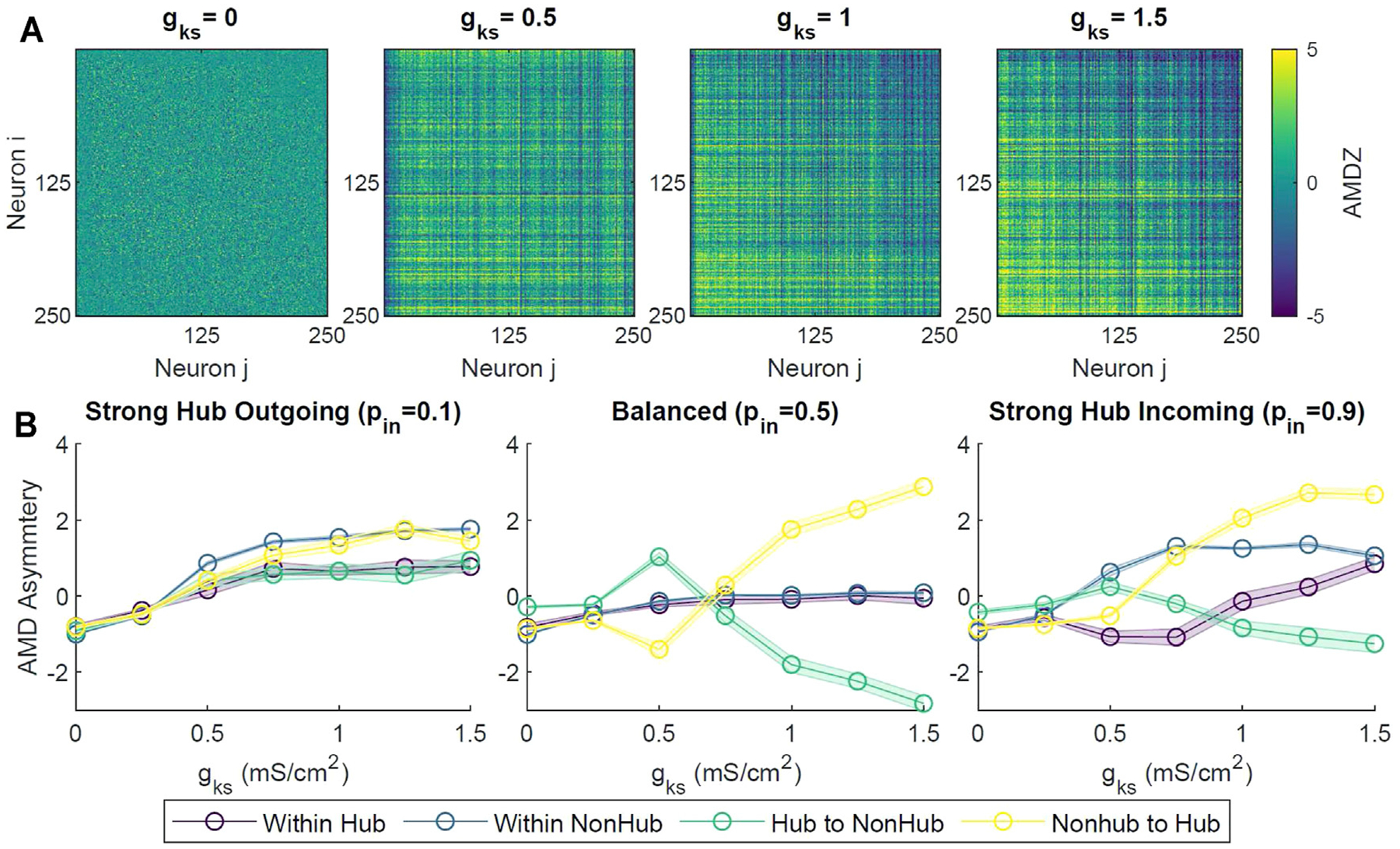
Temporal locking and information flow within the network under different levels of ACh modulation. **(A)** Pairwise Average Mean Distance (AMD) of a sample simulation for different levels of *g*_*ks*_. Neurons were sorted by total degree in descending order, with the highest-degree neuron at position 0. Positive values indicated that neuron *i* typically spiked after neuron *j*, while negative values denoted that neuron *i* activity preceded that of neuron *j*. For *g*_*Ks*_ ≥ 1 mS/cm^2^, high-degree neuron spikes tended to precede those of lower-degree neurons. **(B)** Average AMD Asymmetry scores were calculated from the difference of the AMD matrix and its transpose, then taking the average scores within different groups of neuron connections (Within Hub, Within Non-hub, Hub to Non-hub, Non-hub to Hub). Each value was averaged across 10 simulations across *g*_*Ks*_ levels and network configurations, with shaded bars signifying standard error.

Specifically, we observed that at lower *g*_*Ks*_ values (i.e. high ACh), there were no discernible patterns of locking between neurons across the network (Figure 6A). However, at higher levels of *g*_*Ks*_ (*g*_*Ks*_ = 1, 1.5 mS/cm^2^), the upper triangle of the pairwise AMDZ matrix was primarily negative, suggesting that the spiking activity of neurons with higher degree tended to precede that of neurons with lower degree. The lower triangle of the matrix was predominantly positive, suggesting spiking activity of these neurons generally lagged behind neurons with higher degree. In order to quantify this asymmetric behavior across the network, we calculated an AMD asymmetry matrix by taking the difference between the AMDZ matrix and its transpose (figure not shown). The magnitude of the AMD asymmetry score denoted the consistency in the temporal locking.

To assess the overall causal relationship between the hub and the remaining non-hub neurons, we calculated the average of the AMD asymmetry scores in different sections of the asymmetry matrices (hub to hub, hub to non-hub, non-hub to hub, non-hub to non-hub). Only scores corresponding to existing synaptic connections were averaged. For balanced and strong hub incoming configurations, as *g*_*Ks*_ increased, the average AMD asymmetry scores for the hub to non-hub groups decreased, denoting a reliable leading of the hub activity (Figure 6B). The average AMD asymmetry score increased with increasing *g*_*Ks*_ for the non-hub to hub group, signifying the overall precession of non-hub activity to that of the hub group.

### 2.4 ACh Dependent Structural and Dynamic Network Reorganization

**FIGURE 7 |:**
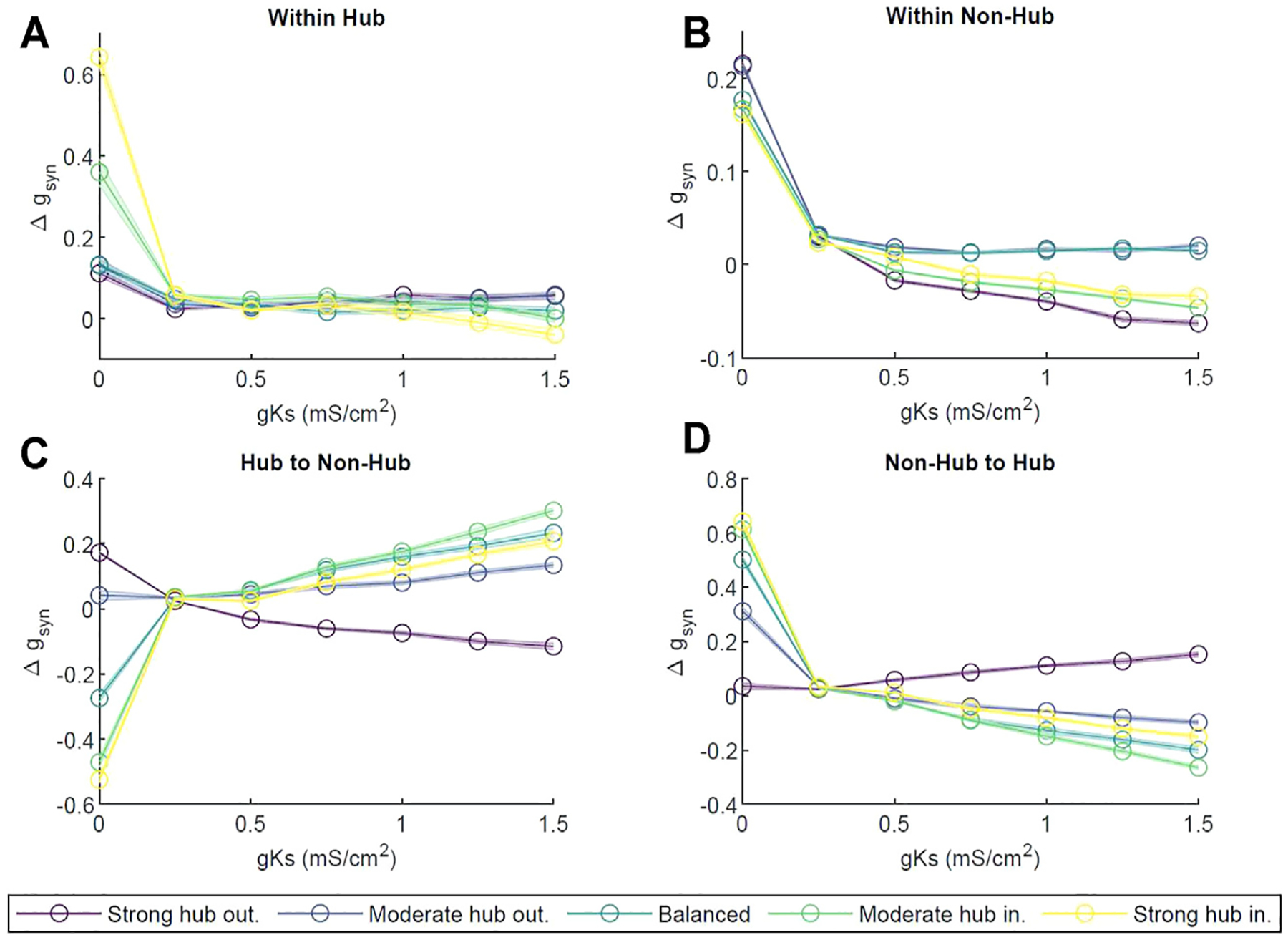
Network reorganization as a function of ACh level. Change in synaptic strength is measured as a function of m-current (*g*_*Ks*_ ∈ [0, 1.5] mS/cm^2^), across 3 s of activity for different network configurations (strong hub outgoing (violet), moderate hub outgoing (dark blue), balanced (teal), moderate hub incoming green) and strong hub incoming (yellow). **(A)** change in synaptic strength measured within the hub; **(B)** within non hub neurons; **(C)** between hub to non hub neurons; **(D)** between non-hub to hub neurons. The changes were normalized by the weight of the initial synapses in the given area. Error bars represent standard error across ten independent trials.

Next, we investigated ACh dependent reorganization of excitatory-only network connectivity, mediated by spike timing dependent plasticity (STDP) (Dan and Poo, 2004). Here, the STDP defined via a fully asymmetric rule with the synapses at which presynaptic neuron fires before the postsynaptic one strengthened and, conversely, the synapses at which presynaptic neuron fires after the postsynaptic one weakened. The amplitudes and time windows for the potentiation and depotentiation are identical (see Section 4). First, we computed the change in synaptic strength Δ*g*_*syn*_ in four areas: the synaptic connections within the hub, within the non-hub neurons, from the hub to the non-hub, and from the non-hub neurons to the hub (see Supplementary Figure S6). In Figure 7A, at *g*_*Ks*_ = 0 mS/cm^2^, synapses strengthened within the hub for all network configurations. The higher the in-degree percentage, the more the synapses strengthened within the hub. As *g*_*Ks*_ increased, the changes in synaptic strength decreased for all *p*_*in*_ values. At *g*_*Ks*_ = 1.5 mS/cm^2^, in the moderate and strong hub incoming configurations, synaptic connections weakened; for the other configurations, they strengthened slightly, within about 5% of zero.

Similarly, within the non-hub group (Figure 7B) at *g*_*Ks*_ = 0 mS/cm^2^, Δ*g*_*syn*_ was significantly positive, indicating a synaptic strengthening for all network configurations. However, for *g*_*Ks*_ > 0 mS/cm^2^, we observed an initial precipitous decline in Δ*g*_*syn*_ and noted that all subsequent changes in *g*_*syn*_ were capped at 5%. While most configurations decreased in connection strength with increasing *g*_*Ks*_, the synapses in the balanced configuration remained unchanged, exhibiting only slight increases.

Thereafter, we analyzed changes in connectivity between the hub and non-hub cells. The synapses from hub neurons to non-hub neurons strengthened as *g*_*Ks*_ increased (Figure 7C) for all network configurations except strong hub outgoing, up to about a 35% increase in the moderate hub incoming configuration at *g*_*Ks*_ = 1.5 mS/cm^2^. We observed a reversed behavior in non-hub to hub synapses (Figure 7D); all network configurations strengthened connections at *g*_*Ks*_ = 0 mS/cm^2^, and most (with the exception of strong hub outgoing) weakened for higher values of *g*_*Ks*_. The magnitude of change in synapses coming into the hub was similar to that of the hub outgoing synapses for a given parameter set.

**FIGURE 8 |:**
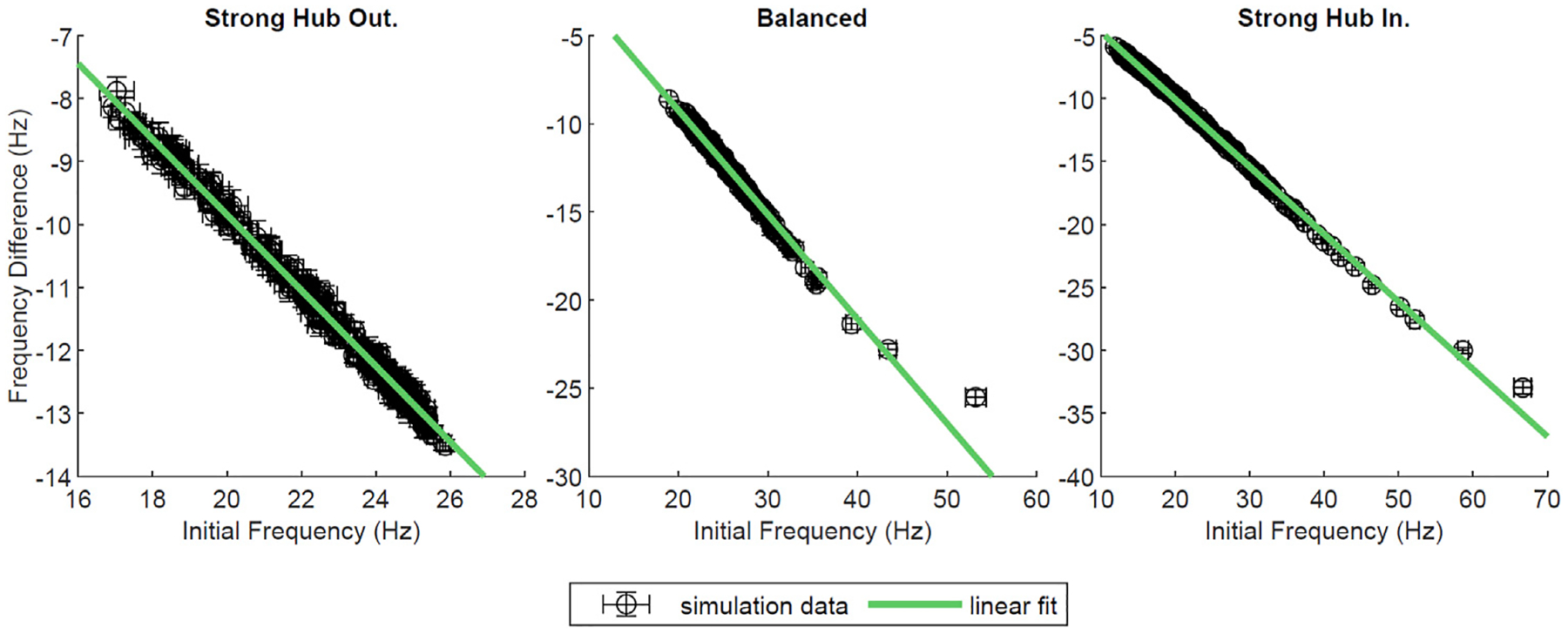
Network wide changes in neural firing frequencies. In this simulation, the standard network was allowed to evolve over 9 s as *g*_*Ks*_ values changed. For the first 3 s, *g*_*Ks*_ was held at 0 mS/cm^2^ and frequency was measured. Then, *g*_*Ks*_ was stepped to 1.5 mS/cm^2^ and synapses were allowed to evolve according to the STDP rule. After 3 s, *g*_*Ks*_ was decreased to 0 mS/cm^2^, STDP was deactivated, and frequency was measured. Here, the initial frequency of each neuron was plotted against the difference of frequency between the first and second *g*_*Ks*_ = 0 mS/cm^2^ segments, in Hz. A line was fit to each dataset; the goodness of fit was *R*^2^ 0.993, 0.983, 0.997, from left to right. This measure was plotted for various network configurations, and error bars denote a standard error across ten trials.

In most of our network configurations (except strong hub out), NREM like (low-ACh) states allow for the overall strengthening of connections from the hub to non-hub neurons, and conversely, the weakening of the connections from non-hub to hub cells. The connections between hub and within non-hub neurons undergo much less pronounced reorganization. Therefore, we wanted to assess the effects of network reorganization during a low-ACh state (sleep) on the network dynamics recorded during a high-ACh state (wake). To this end, we compared network activity patterns during three-second segments of simulation at *g*_*Ks*_ = 0 mS/cm^2^ (high ACh state) before and after a three-second segment of STDP at *g*_*Ks*_ = 1.5 mS/cm^2^ (low ACh state). We were interested in changes in neuronal firing frequency between the two *g*_*Ks*_ = 0 mS/cm^2^ segments, as such a change was observed experimentally (Clawson et al., 2018). Firing frequency was measured across the two *g*_*Ks*_ = 0 mS/cm^2^ segments, and the difference between them was plotted against the frequency during the first *g*_*Ks*_ = 0 mS/cm^2^ segment (Figure 8). In all network configurations (i.e. strong hub-out, balanced and strong hub-in), the neurons slowed their firing rates following the low-ACh segment. Furthermore, each plot displayed a linear downward trend, implying that the neurons firing at a high frequency in the first *g*_*Ks*_ = 0 mS/cm^2^ segment were more likely to experience a larger negative change in frequency. After performing a linear regression on each dataset, we found slopes for all configurations to be between −0.5 and −0.6 (R^2^ > 0.98).

The fact that all of the neurons here exhibit decline in firing frequency even though the hub to non-hub connections are strengthened, is due to the hub cells being very few in number, while the observed strengthening is offset by another modes of reorganization, as described above.

### 2.5 Hub Removal

**FIGURE 9 |:**
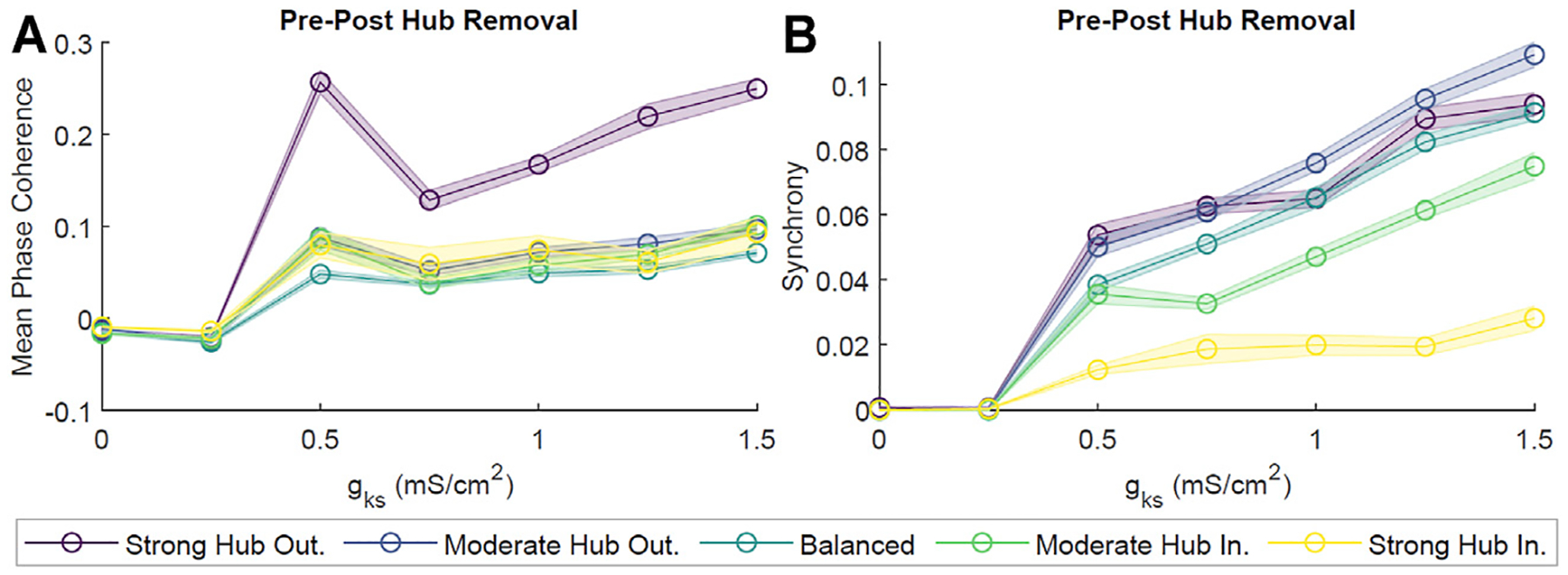
Effects of neuronal hub removal on ACh-modulated network dynamics. To model hub removal, we first performed our standard simulation for 2 s. Then, we set all synaptic connections associated with the hubs (incoming and outgoing) to zero, effectively removing the influence of these neurons from the network, and allowed the simulation to evolve for two more seconds. Subsequently, we calculated the difference in the average Mean Phase Coherence **(A)** and Synchrony **(B)** between remaining neurons pre- and post-hub removal for different levels of *g*_*Ks*_ and network configurations. Averages were obtained from 10 independent simulations per parameter set and shaded bars represents standard error of the mean. Hub removal led to a decrease in Mean Phase Coherence and Synchrony.

Given that hub neurons were in direct correspondence to a large fraction of the network, they sat at a critical position for potentially mediating the dynamics within the network. In order to assess the role of hub neurons in cholinergically-modulated network dynamics, we removed these neurons from our scale-free networks and calculated the change in network coherence and synchrony. We observed effectively no change in pattern formation for *g*_*Ks*_ ≃ 0 mS/cm^2^, as both pre- and post-hub removal MPC and Synchrony were low (Figure 9). However, as *g*_*Ks*_ increased, the difference in pre- and post-hub removal dynamics was more noticeable. This was especially true for the MPC of the hub–outgoing configurations (Figure 9A). The differences in Synchrony between pre- and post-hub removal were significantly smaller (Figure 9B). This indicated that the remaining connections, together with high *g*_*Ks*_, could support synchronous bursting while the stability in phase ordering was significantly affected. For example rasterplots, please see (Supplementary Figure S7).

## 3 DISCUSSION

ACh levels throughout the forebrain have been known to vary with different wake/sleep states, with high cholinergic tone during wake and REM and low cholinergic tone during SWS (Jasper and Tessier, 1971; Marrosu et al., 1995; Watson et al., 2012). However, its exact role on network dynamics across these states is lesser known. Here, we concentrate on cholinergic modulation of cell excitability via muscarinic receptors. These receptors regulate conductance of the slow hyperpolarizing M-type potassium protein channel (*g*_*Ks*_), modulating the neuronal membrane excitability. When M-type potassium currents are low, the neuron exhibits a steep input-frequency (I-F) curve and a Type I phase response curve (PRC) characteristic of an integrative dynamical system (Ermentrout, 1996; Fink et al., 2011; Rich et al., 2016). These neurons simply integrate any input signal towards firing an action potential, which makes the synchronization of neuronal activities difficult given the heterogeneity of the synaptic connections and input levels. With higher levels of M-type potassium current, the model exhibits a flattened I-F curve, leading to similar firing frequencies across the network and a Type II PRC that responds preferentially to specifically timed inputs, facilitating firing pattern synchronization in connected neurons. This observation aligns with studies where selective electrical stimulation of cholinergic neurons and pharmacological stimulation of muscarinic acetylcholine receptors hindered slow oscillating rhythmic activity characteristic of slow wave sleep state and promoted a more tonic firing pattern typical in REM sleep and wakeful states (Steriade et al., 1993; Baghdoyan and Lydic, 1999).

We are specifically interested in pattern formation within networks with highly heterogeneous connectivity topologies, e.g. scale-free networks (Albert et al., 2000; Bollobás et al., 2001). This power law degree distribution creates networks largely consisting of nodes with sparse connections and a small group of nodes with an extremely high number of connections (hubs). Additionally, within this framework, we also consider network structures that 1) favor connections going into the hubs (hub incoming), 2) have relatively equal distributions of connections beginning and terminating at the hubs (balanced), or 3) are biased towards connections predominantly emanating from the hubs (hub outgoing).

Scale-free networks are embedded within the frameworks of many complex systems such as the World Wide Web, social networks, metabolic organization, and protein-protein interaction hierarchy in many microorganisms such as *E. coli* (Albert et al., 2000; Jeong et al., 2000; Jeong et al., 2001). Many studies have discovered scale-free topologies within the brains of animal species including mice, rats, cats, and primates, including humans (van den Heuvel et al., 2016; Zamora-López et al., 2010; Harriger et al., 2012). Activity-based functional connectivity measures and myelin tract tracing-based structural connectivity studies have identified hub-like regions in areas including the hippocampus, thalamus, basal ganglia structures, and various regions of the neocortex (Eguiluz et al., 2005; Bonifazi et al., 2009; Li et al., 2010; Van Den Heuvel and Sporns, 2011). Furthermore, a recent study exploring the structure of the mouse connectome sorted different scale-free topologies within the brain into categories based on the overall direction of connections with respect to these hubs (Coletta et al., 2020).

We quantified pattern formation in the network via measurement of frequency profiles of individual neurons, the tendency of neurons to generate population burst via network-averaged pairwise cross-correlation measure, and stability of directional firing patterns between individual neurons. We also investigated network reorganization driven through dynamics during low-cholinergic states.

We show that at low *g*_*Ks*_ levels corresponding to high-ACh modulation, neurons fire at highly discrepant frequencies, with hubs firing significantly faster than the non-hub neurons. We also found no discernible network-wide temporal patterns of firing activities. However, as we increased *g*_*Ks*_, the firing patterns throughout the network became increasingly synchronous (Figure 2). Our results corroborated with *in vivo* studies that associated wake states with highly heterogeneous neuronal spiking frequencies and asynchronous neuronal activity, whereas slow wave sleep was characterized by periods of quiescence and shorter periods of synchronous firing (Steriade et al., 2001; Vyazovskiy et al., 2009).

Subsequently, we explored how scale-free topologies with different in-degree distributions affect cholinergically-modulated network dynamics. All of our network configurations yielded similar behaviors of increasing network activity, coherence, and synchrony as a function of *g*_*Ks*_ (Figures 2B–E). However, individual MPC and CC scores differed across network configurations. In most cases, the balanced network configuration achieved the highest MPC and CC. As we shifted the network configuration away from balanced in either direction, the global MPC and CC decreased. These results can be assumed due to the balanced incoming and outgoing connections allowed for sufficient communication between the hub and non-hub neurons to synchronize the network. It has been implicated in literature that hubs not only act as signal generators, but also as means of amplifying signals from non-hub subnetworks to the rest of the network, thereby suggesting the importance of a well-balanced, bi-directional communication between the hub and the rest of the network (Jahnke et al., 2014).

In the strong hub outgoing configuration, the synchrony within the hub was slightly lower than that of the rest of the network. This may have been due to a bottlenecking effect, where the hub was not receiving the input necessary to tightly synchronize itself, as most of its connections are directed outwards. In the strong hub incoming configuration, synchrony within the hub was significantly higher than that of the non-hub group. This may once again be due to a bottleneck hindering the hub from sending output to synchronize the rest of the network. Even so, the trend of increasing synchrony with decreasing cholinergic modulation persisted for all network configurations.

We also assessed the robustness of our results against a range of synaptic connectivity strengths and noisy external inputs. Increasing network wide connectivity strength increased activity, coherence, and synchrony, while increasing the amplitude of a random noisy external input led to a decrease in coherence and synchrony (Figures 3, 4). Synchrony and coherence increased as a function of *g*_*Ks*_ when the connectivity strength between neurons was strong and the amplitude of the noisy input was low.

We wanted to see if this trend was robust against inhibitory input. Since, inhibitory neurons constitute about 10–15% of the hippocampal population (Pelkey et al., 2017), we directly compared results obtained for excitatory-only network with those obtained from the networks having 10% of inhibitory neurons. In addition, we included comparisons of coherence and synchrony within hub and non-hub cells for networks consisting of 10, 15, 20% of inhibitory cells. We observed that coherence and synchrony across the network were lower than those of a purely excitatory network. We suspected that random and sparse synapses of the inhibitory neurons caused an unequal distribution of inhibitory signals to be broadcast to the network, thereby decreasing overall network synchrony and coherence. Even so, we were able to produce a similar behavior of increasing network synchrony and coherence with decreasing cholinergic modulation.

While new information is encoded during wake, SWS is essential for many types of memory consolidation (Rasch and Born, 2013; Hasselmo, 1999). This function is mediated by SWS-associated changes in cholinergic modulation; preventing the SWS-dependent reduction in ACh transmission after learning has adverse consequences for consolidation of declarative memory tasks (Gais and Born, 2004; Haam and Yakel, 2017). However, the mechanism by which changes in ACh modulation influence this process is not well understood. Since it is widely accepted that the neural correlate of learning and memory is most likely encapsulated within the ever-changing synaptic weights between neurons, we wanted to assess how levels of ACh affect network-wide STDP based learning (Stuchlik, 2014; Hebb, 1949; Dan and Poo, 2004). Generally, when interrogating all existing connections together, at *gKs* = 0 mS/cm^2^ (high ACh), we observed a global increase in *g*_*syn*_, (Figure 7). Conversely, at *g*_*Ks*_ ≥ 1 mS/cm^2^ (low ACh), we saw a moderate decrease in *g*_*syn*_. This result generally aligns with the synaptic homeostasis hypothesis, which posits a general weakening of synapses during sleep to counteract the global strengthening of synapses during wakefulness (Tononi and Cirelli, 2014), (Bushey et al., 2011; Maret et al., 2011). Our previous modeling work also showed STDP-mediated increases in synaptic weights in Type I neurons as well as a decrease in synaptic weights in Type II neurons (Fink et al., 2013).

Critically, however, we saw differential behavior of connectivity between subgroups within the network in our simulations. Specifically, at *g*_*Ks*_ ≥ 1 mS/cm^2^ (low ACh), we saw an inverse change in synaptic connectivity between the hub and non-hub groups (Figure 7). We observed an increase in the synaptic conductance from hub neurons to the rest of the network, accompanied by a decrease in the synaptic conductance of the synapses from the non-hub neurons to the hub. Because STDP dictates simultaneous strengthening of some synapses and weakening of others, this elucidated a strong consistent leading and lagging of neuronal spiking activity between the hub and non-hub neurons. In particular, hub neurons consistently fired action potentials before non-hub neurons, while non-hub neurons reliably spiked after the connecting hubs. This behavior was indeed predicted in our temporal locking (AMD) analysis in simulations without STDP, showing a similar inverted directionality between hubs and non-hubs (Figure 6).

These results indicate a potential role of acetylcholine in regulating memory consolidation via a STDP mechanism. Our results showed that high levels of cholinergic modulation, indicative of a wakeful state, led to a strengthening of synapses predominantly within the hub cells, from no-hub cells to hub cells, and to lesser degree between the non-hub cells At the same time, low levels of cholinergic modulation representative of a SWS state led to a preferential strengthening of synapses from the hub to the rest of the network.

To further test how low-ACh network reorganization affects waking network dynamics, we performed additional simulations where the sleep reorganization phase was preceded and followed by a high-ACh phase. We observed that the global frequency profile changed significantly between the pre- and post-low-ACh states. Namely, the neurons that initially fired with the highest frequency during high-ACh state, that preceded network reorganization during low-ACh(i.e. hub cells), exhibited the largest drop in their firing frequency during high ACh state following this reorganization. Conversely, the neurons that initially fired at the lowest frequencies (i.e. non-hub neurons with the least number of connections) exhibited a smaller magnitude of decrease in frequency in high-ACh state following the low-ACh state. Various *in vivo* studies have shown that SWS-mediated frequency dependent changes in firing rates within the visual cortex of mice led to the homogenization of spiking frequency of hippocampal neurons (Clawson et al., 2018; Durkin and Aton, 2019; Miyawaki et al., 2019).

Our results further indicate that in a high-ACh state, the hub network is predominantly strengthened via hub-hub connections and non-hub to hub connections with additional strengthening taking place from within non-hub network. This is indicative of memory reinforcement predominantly in hub network with some input coming from non-hub cells. Conversely, in low ACh states, only hub to non-hub connectivity is strengthened, while all other connections remain unchanged or weaken. This, in turn, is indicative of information transfer from the hub cells to non-hub neurons.

In the presented network the hub population was very sparse as compared to non-hub group. Therefore, the increase of excitatory input from the hub cells was offset by the decrease in of the excitation coming from other network regions. Hence, we observed an overall decrease of firing across the cell populations. This phenomena is consistent with the synaptic renormalization hypothesis (Tononi and Cirelli, 2014), postulating overall depotentiation of synapses during sleep. However, we also show that this weakening can be highly selective.

Based on these results, if we consider that hub neurons could be playing a central role in regulating sensory input to the network, we hypothesize that this state-dependent network reorganization mechanism during wake may be responsible for the initial formation of a memory backbone within the hub network. Then during the subsequent SWS, the recruitment of new neurons (i.e., those initially outside the hub) into the memory engram provides the basis for systems consolidation—i.e., expansion of memory traces throughout the network (Roach et al., 2018; Puentes-Mestril et al., 2019). Namely, this phenomenon could correspond to the recruitment of initially less active neurons into the memory engram within hippocampal networks, and/or mediate information transfer between the hippocampus and neocortex, which could provide an explanation as to the experimentally observed increased correlation between hippocampal and neocortical neurons during NREM (Sirota et al., 2003).

Finally, given the hub’s unique position as the most heavily connected neuronal group in the network, we suspected that the hub must play a crucial role in mediating the activity of the network as a whole. It is well known that while scale-free networks are relatively resilient against random failures at particular nodes, disruption of network hubs will lead to catastrophic network failure (Albert et al., 2000). For example, simulations knocking out a large number of highly centralized proteins led to lethality in a model of *Saccharomyces cerevisiae*, and the removal of strong functionally-connected neuronal tissue during glioma resection correlated to higher likelihood of postoperative language and auditory deficits (Jeong et al., 2001; Lee et al., 2020). In this study, we saw a significant decrease in overall network coherence and synchrony following the removal of hub neurons (Figure 9). This effect was greater in network configurations where hubs had high outgoing to incoming connection ratios, with the strong hub outgoing configuration displaying the highest decrease in post-hub removal coherence and synchrony. This result corroborates with previous work in scale-free networks of leaky integrate-and-fire neurons, where removal of hub neurons led to arrested network synchrony (Luccioli et al., 2014). Other studies have indicated the importance of hubs in orchestrating and maintaining signal synchronization throughout the network (Gómez-Gardeñes et al., 2010; Jahnke et al., 2014).

Overall, our results provide insight into the possible role of ACh in dynamical and structural reorganization in highly heterogenous networks. We demonstrated that high ACh modulation representative of wake causes a high frequency, asynchronous firing pattern throughout the network associated with global synaptic strengthening, while low ACh modulation representative of SWS causes a low-frequency synchronous firing pattern and general synaptic weakening. Additionally, we showed that the arrangements of neurons in a scale-free topology gave rise to specific behaviors within the hub and non-hub groups, where the hub played a prominent role in synchronizing the remainder of the network during low ACh modulation. These results shed light on the dynamical mechanisms underlying memory consolidation during sleep.

## 4 METHODS

### 4.1 Neuron Model

The biophysical model of each neuron was based on a Hodgkin-Huxley formalism (Hodgkin and Huxley, 1952), modified to include an M-type potassium current (Stiefel et al., 2009; Fink et al., 2013). The change in voltage across the cell membrane was given by

(1) $$C\frac{dV}{dt}=-g_{Na}m_{\infty }^{3}(V)h\left(V-E_{Na}\right)-g_{Kdr}n^{4}\left(V-E_{K}\right)-g_{Ks}z\left(V-E_{K}\right)-g_{L}\left(V-V_{L}\right)+I^{\text{drive}}+I^{\text{noise}}-I^{syn}$$

Here, *C* = 1.0 *μ*F/cm^2^ was the membrane capacitance, *V* was in millivolts, and *t* was in milliseconds. Setting *g*_*Ks*_ = 0 mS/cm^2^ modeled a high concentration of acetylcholine, while *g*_*Ks*_ = 1.5 mS/cm^2^ indicated a low concentration (Fink et al., 2013).

Other ionic conductance values were constant across all simulations: the sodium channel conductance, *g*_*Na*_ = 24.0 mS/cm^2^; the delayed rectifier potassium conductance, *g*_*Kdr*_ = 3.0 mS/cm^2^; and the leak conductance, *g*_*L*_ = 0.02 mS/cm^2^ (Fink et al., 2013). The reversal potential for each ion was also held constant: the sodium reversal potential *E*_*Na*_ = 55.0 mV, the potassium reversal potential *E*_*K*_ = −90.0 mV; and the leak, *E*_*L*_ = −60 mV (Fink et al., 2013). *I*^*drive*^ was a constant externally applied current; this value was chosen from the frequency-current curve such that it was the highest applied subthreshold current. *I*^*noise*^ was a 2 ms current pulse of amplitude 0.7 *μ*A/cm^2^, and occurred with a probability of 0.02 at each time step, generating noise at an average frequency of 200 Hz. *I*^*syn*^ was the synaptic current received by a given neuron.

The sodium current was governed by the steady state activation function:

(2) $$m_{\infty }(V)=\frac{1}{1+e^{\frac{-V-30.0}{0.5}}}.$$

The sodium current inactivation gating variable, *h*, was described by:

(3) $$\frac{dh}{dt}=\frac{h_{\infty }(V)-h}{\tau_{h}(V)},$$

where the steady-state activation was described by

(4) $$h_{\infty }(V)=\frac{1}{1+e^{\frac{V+53.0}{7.0}}}$$

and the timescale variable was

(5) $$\tau_{h}(V)=0.37+2.78\frac{1}{1+e^{\frac{V+40.5}{6.0}}}.$$

The delayed rectifier potassium current was gated by *n*, where

(6) $$\frac{dn}{dt}=\frac{n_{\infty }-n}{\tau_{n}(V)}.$$

The steady-state activation was given by

(7) $$n_{\infty }(V)=\frac{1}{1+e^{\frac{-V-30.0}{10.0}}}$$

and the time variable was

(8) $$\tau_{n}(V)=0.37+1.85\frac{1}{1+e^{\frac{V+27.0}{15.0}}}.$$

Finally, the slow, low threshold M-type potassium current was gated by *z*, where

(9) $$\frac{dz}{dt}=\frac{z_{\infty }(V)-z}{75.0},$$

with

(10) $$z_{\infty }(V)=\frac{1}{1+e^{\frac{-V-39.0}{5.0}}}.$$

This system of equations was solved using a fourth-order Runge-Kutta numerical scheme in Matlab with time step *dt* = 0.1 ms. Initial voltage conditions were randomly chosen from [−70, 0] mV, and were zero for all gating variables except for *h*, where *h* (0) = 1. Unless otherwise noted, each simulation evolved over 2 s. Each result was simulated independently ten times with new initial conditions and a new connectivity matrix.

### 4.2 Network Simulation

The network contained a total of 250 neurons. In the majority of simulations, we investigated an excitatory-only network. If the network was mixed, it contained 225 excitatory cells and 25 inhibitory cells. In this case, the excitatory neurons formed a scale-free network, while connections between inhibitory neurons were random, with every neuron having approximately the same number of connections.

The scale-free network structure was constructed using the Barabasi-Albert Linearized Chord Diagram algorithm (Bollobás et al., 2001). First, we began with an empty graph with no nodes, denoted $G_{1}^{0}$. Then, given a graph $G_{1}^{u-1}$, we generated graph $G_{1}^{u}$ by adding a new node, *v*_*u*_, to some existing node, defined *v*_*i*_ (Bollobás et al., 2001). The probability *p* of a given node in $G_{1}^{u-1}$ being chosen was defined:

(11) $$p=\{\begin{array}{ll} \frac{k_{i}}{2u-1} & 1\leq i\leq u-1\\ \frac{1}{2u-1} & u=1 \end{array}$$

Here, *k*_*i*_ was the degree of node *i*, and *u* was the total number of nodes (Bollobás et al., 2001). No self-connections or multi-connections were allowed; connections were initially bidirectional. The algorithm was executed 15 times so that the average degree of the nodes was 15, and the strength of the connection was initialized at *w*_*ij*_ = *w*_*ji*_ = 0.04 mS/cm^2^. Once the network was constructed, direction was assigned to each connection according to a probability *p*_*in*_ ∈ {0.1, 0.3, 0.5, 0.7, 0.9}. First, neurons were arranged in order of decreasing degree, such that the neuron with label 1 had the highest degree. Then, iterating through half of the symmetrical connectivity matrix, a random number *r* ∈ [0, 1] was generated for each existing connection between neurons *i*, *j*. Then, we set

(12) $$\{\begin{array}{ll} w_{ij}=0 & r>p_{in}\\ w_{ji}=0 & r<p_{in}. \end{array}$$

Thus, we determined the proportion of incoming to outgoing connections in the hub neurons (see Supplementary Figure S2), where the hub was defined as the top 10% of neurons by total degree, or the 25 neurons with the most connections (see Supplementary Figure S6). For values of *p*_*in*_ ∈ {0.1, 0.3, 0.5, 0.7, 0.9}, networks were referred to as “strong hub outgoing,” “moderate hub outgoing,” “balanced,” “moderate hub incoming,” and “strong hub incoming,” respectively.

Neuron *i* received a synaptic current from presynaptic neuron *j* at times *t*_*jk*_, when *V*_*j*_ > 0 mV. The synaptic current transmitted from *j* to a postsynaptic neuron *i*, where *t* ≥ *t*_*jk*_, was given by

(13) $$I_{ij}^{syn}=w_{ij}\left(e^{-\frac{t-t_{jk}}{\tau_{d}}}-e^{-\frac{t-t_{jk}}{\tau_{r}}}\right)\left(V-E_{syn}\right).$$

Here, *τ*_*d*_ 0.5 ms, *τ*_*r*_ 0.2 ms, and *w*_*ij*_ was the strength of the synapse from *j* to *i* (adapted from (Roach et al., 2018)).

For excitatory synapses, *E*_*syn*_ = 0 mV (Fink et al., 2013), while for the inhibitory neurons *E*_*syn*_ = −75 mV. The connection strength, or synaptic conductance, between inhibitory neurons was set to 0.01 mS/cm^2^, and remained 0.04 mS/cm^2^ elsewhere. The total synaptic current given to neuron *i* at each time step was described by

(14) $$I_{i}^{syn}=\underset{j}{\sum }I_{ij}^{syn},j\in w_{i},$$

where *w*_*i*_ was the set of all neurons presynaptic to *i* (Fink et al., 2013).

### 4.3 Spike Timing Dependent Plasticity

In the simulations that included STDP (Kempter et al., 1999; Fink et al., 2013), excitatory synaptic connections were initially weighted equally at *w*_*ij*_ = *w*_0_ = 0.04 mS/cm^2^ for all neurons *i*, *j*. The change in synaptic strength between postsynaptic neuron *i* and presynaptic neuron *j* was given by

(15) $$\Delta w_{ij}=\{\begin{array}{cc} \frac{-\left|\Delta t\right|}{A_{L}e^{\tau_{\text{STDP}}}} & \Delta t>0\\ \frac{-\left|\Delta t\right|}{A_{L}e^{\tau_{\text{STDP}}}} & \Delta t<0 \end{array}$$

where Δ*t* was the difference between the spike time of postsynaptic neuron *i* and presynaptic neuron *j*. In other words, if neuron *j* spiked before neuron *i*, the connection from *j* to *i* strengthened. *A*_*L*_ = 0.002 mS/cm^2^ was a constant describing the maximal synaptic change due to learning, and *τ*_*STDP*_ = 10 ms was a constant describing the decay rate of the weight change over time. Any one synapse was bounded ∈ [0, *w*_max_], where *w*_max_ = 2 · *w*_0_, and synapses that did not exist at the beginning of the simulation were not created. Learning only occurred if the spikes of neurons *i*, *j* were within a window of 40 ms.

All parameters of the model are summarized in (Table 1). The network simulation software is also available at: https://github.com/J4KLin/scaleFreeGksNeuronalNetwork.

### 4.4 Measures

We used Mean Phase Coherence (MPC) to quantify average phase locking between individual cells in the network (Mormann et al., 2000). The measure was calculated pairwise between all neurons. A mean phase coherence of zero indicated asynchronous spiking as defined by a non-constant phase, while a value of one described complete phase locking (Mormann et al., 2000; Fink et al., 2013). It is important to note that a high MPC between neurons *i*, *j* indicates neuron *j* spikes at a constant phase *relative to neuron i*, not necessarily at the same time as neuron *i* (Fink et al., 2013).

Consider a pair of neurons *i* and *j*. The pairwise coherence between these two neurons was defined by:

(16) $$\sigma_{i,j}=\left|\frac{1}{N}\overset{N}{\underset{k=1}{\sum }}e^{i\phi_{k}}\right|,$$

where

(17) $$\phi_{k}=2\pi \left(\frac{t_{j,k}-t_{i,k}}{t_{i,k+1}-t_{i,k}}\right).$$

Here, *t*_*j*,*k*_ was the time of the k^*th*^ spike of neuron *j*, *t*_*i*,*k*_ was the time of the spike of neuron *i* that was just before *t*_*j*,*k*_, *t*_*i*,*k*+1_ was the spike time of neuron *i* that was the just after *t*_*j*,*k*_, and *N* was the number of spikes of neuron *j*. The mean phase coherence, *σ*_*i*,*j*_ was calculated for every pair of neurons {*i*, *j*} in the network. These pairwise measures were averaged across the whole network. MPC was calculated only during the second half of the simulation, in order to avoid transients due to initial conditions in the first half.

To asses the degree of synchrony, we calculated zero-lag cross correlation by first convolving the spike train *S*_*i*_ of given neuron *i* with a Gaussian of width *σ* = 1 ms. This convolved spike train was rescaled by subtracting its mean, resulting in continuous trace $S_{i}^{*}$. The pairwise cross correlation was calculated as

(18) $$C_{ij}=\frac{S_{i}^{*}\cdot S_{j}^{*}}{\sqrt{\left(S_{i}^{*}\cdot S_{i}^{*}\right)\left(S_{j}^{*}\cdot S_{j}^{*}\right)}}.$$

The average network cross correlation was calculated as a mean of the pairwise cross correlation values for all neurons *i*, *j*, where *i* ≠ *j*.

To quantify the average spike ordering between pairs of spiking neurons, a metric based on the average minimal distance (AMD) was used (Wu et al., 2018). The pairwise AMD between two neurons *i*, *j* was given by the mean difference of the time of each spike *k* in spike train of neuron *i*, *S*_*i*_, to the most recent preceding spike in the spike train of neuron *j*, *S*_*j*_ (Wu et al., 2018). Specifically, the pairwise AMD was given by

(19) $$AMD_{ij}=\frac{1}{N_{i}}\underset{k}{\sum }\Delta t_{k}^{j},$$

where *N*_*i*_ was the number of spikes in spike train *S*_*i*_ and $\Delta t_{k}^{i}$ was the temporal distance between a spike *k* in *S*_*i*_ to the nearest event in *S*_*j*_ (Wu et al., 2018).

In order to quantify the magnitude of temporal locking within the network, an asymmetry score was calculated. Let *L* be the length of the interspike interval of the spiketrain *S*_*i*_. Then the first and second moments (*μ*_1_ and *μ*_2_, respectively) for the spiketrain *S*_*i*_ were given by

(20) $$\mu_{1}=\frac{1}{2T}\underset{L}{\sum }L^{2},\mu_{2}=\frac{1}{3T}\underset{L}{\sum }L^{3},$$

where *T* was the total time of the spike train *S*_*i*_ (ms) (Wu et al., 2018). The moments were used to derive the mean and standard deviation of the minimal distance with respect to *S*_*i*_ where the mean *μ = μ*_1_, the first moment, and the standard deviation $\sigma =\sqrt{\mu_{2}-\mu_{1}^{2}}$ (Wu et al., 2018). Then, the Z-score was calculated, where the Z-score $Z_{ij}=\frac{AMD_{ij}-\mu_{i}}{\sigma_{i}}$. Then, the Z-score was used to calculate AMD asymmetry, given by *Z*_*ij*_ − *Z*_*ji*_.

Network reorganization was quantified by the change in synaptic strength across the simulation. These changes were measured in different areas of the network: the hub, the non-hub, synapses presynaptic to the hub (non-hub to hub), and synapses postsynaptic to the hub (hub to non-hub). Here, the hub was defined as the top 10% of neurons by total degree; non-hub neurons were the remaining neurons. These areas are described visually in the schematic at (Supplementary Figure S6). In each of these zones, the magnitude of change was given by

(21) $$\Delta g_{syn}=\frac{\sum_{ij}\Delta w_{ij}}{A\cdot w_{0}},$$

where *w*_0_ was the initial synaptic strength, {*ij*} was the set of all synapses in a given region of the network, Δ*w*_*ij*_ was the change in the magnitude of the synaptic strength of a given connection, and *A* was the number of total synapses in the given region.

In addition, we have measured spectral properties of Local field potential (LFP; Supplementary Figure S3). For every network simulation we summed voltages of all (excitatory) neurons in the network, normalized the cumulative signal and performed FFT. The results shown are averaged over 10 simulations each.

### 4.5 Measurement of Effects of Network Reorganization During Sleep on Waking Dynamics

Finally, to measure the effects of network reorganization during sleep on waking dynamics and compare them with experimental findings (Clawson et al., 2018), the network was allowed to evolved for 9 s total under different acetylcholine conditions. During the first 3 seconds, *g*_*Ks*_ = 0 mS/cm^2^ and the weight of synaptic connections was kept constant. The frequency of each neuron was measured across the latter 2 seconds of this segment in order to avoid transients due to initial conditions. In the next 3 seconds, *g*_*Ks*_ was stepped to 1.5 mS/cm^2^ and synaptic weights were allowed to evolve according to the STDP learning rule. In the final 3 seconds, synaptic weights were no longer allowed to change and *g*_*Ks*_ was returned to 0 mS/cm^2^; frequency was measured as above.

## Supplementary Material

supplementary info

## Figures and Tables

**TABLE 1 |:** Summary of parameters used in the simulations.

Parameter names	Parameter descriptions	Values
Simulation		
Dt	integration time steps	0.1 ms
U	# of neurons	250
Network connectivity		
Z	# of iterations (loops) used to generate the Scale free network using the Linearized Chord Diagram algorithm	15
p_in_	percentage of hub in-degree (connectivity)	0.1 (strong hub out), 0.3 (moderate hub out), 0.5 (balanced), 0.7 (moderate hub in), 0.9 (strong hub in)
Spike timing dependent plasticity (STDP)		
A_L_	STDP amplitude/spike event	0.0002 mS/cm^2^
τ_STDP_	STDP decay constant	10 ms
Neuron model		
C	membrane capacitance	1 mF/cm^2^
V_0_	initial voltage	random from [−70, 0] mV
g_Kdr_	delayed rectifier potassium conductance	3 mS/cm^2^
G_Na_	sodium conductance	24 mS/cm^2^
G_L_	leak conductance	0.02 mS/cm^2^
G_Ks_	m-current conductance	0–1.5 mS/cm^2^
E_Na_	Sodium reversal potential	55 mV
E_L_	Leak potential	−60 mV
E_K_	Potassium reversal potential	−90 mV
I^drive^	constant applied current	Chosen from frequency- current curve for each gKs such that the applied current is just subthreshold [mA/cm^2^]
I^noise^	random 2 ms current pulse with 2% probability at each time step (unless otherwise posted)	0.7 mA/cm^2^ (unless otherwise posted)
Synaptic connectivity		
g_Syn_ (excitatory connections)	synaptic conductance	0.04 mS/cm^2^ (unless otherwise posted)
g_Syn_ (inhibitory connections)	synaptic conductance	0.01 mS/cm^2^
E_syn_ (Excitatory)	excitatory synapse reversal potential	0 mV
E_syn_ (inhibitory)	inhibitory synapse reversal potential	−75 mV
τ_d_	synaptic delay constant	0.5 ms
τ_r_	synaptic rise constant	0.2 ms

## Data Availability

The datasets presented in this study can be found in online repositories. The names of the repository/repositories and accession number(s) can be found below: https://github.com/J4KLin/scaleFreeGksNeuronalNetwork.
