# Supplementary material for: Dynamical Mechanism Underlying Scale-Free Network Reorganization in Low Acetylcholine States Corresponding to Slow Wave Sleep
Source: Front Netw Physiol. 2021 Oct 25;1:759131. doi: 10.3389/fnetp.2021.759131 (PMC9249096; doi:10.3389/fnetp.2021.759131)
Supplement: Supplementary file 1 [file DataSheet1.PDF]

Supplementary Material

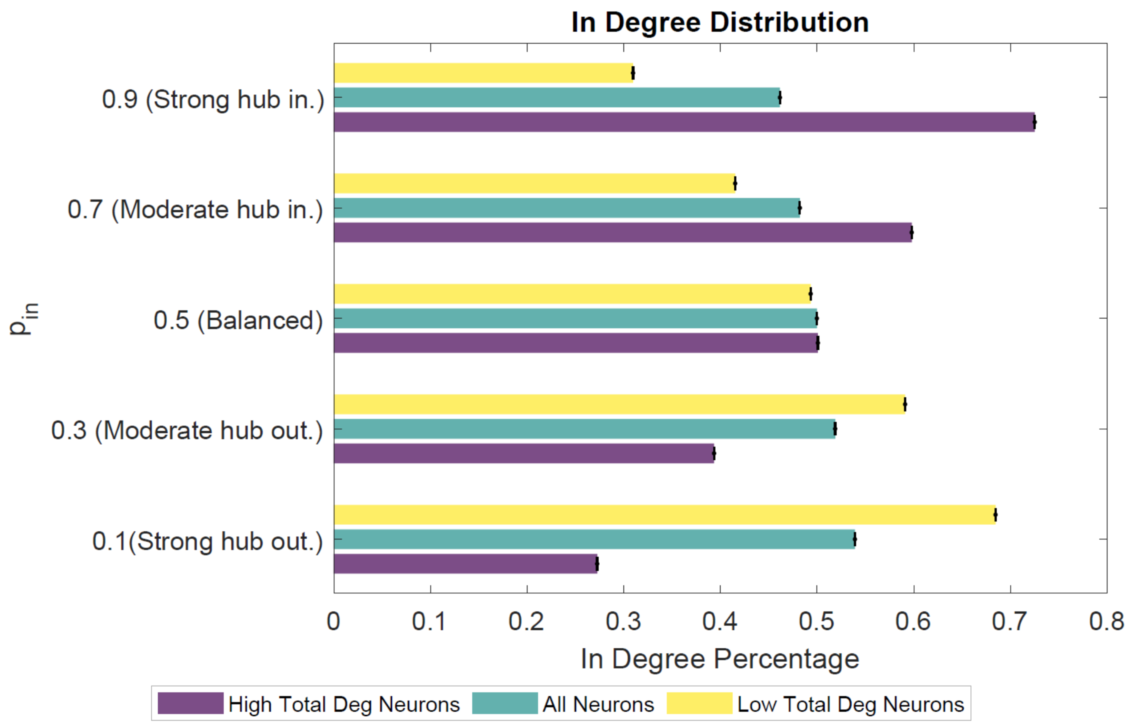

Figure S1. Different scale-free network configurations result in different proportions of low- and high-degree neurons. We created five network configurations by applying a probabilistic synapse direction swapping algorithm using probability values (p_{in}) of 0.1, 0.3, 0.5, 0.7 and 0.9, each corresponding to our strong hub outgoing, moderate hub outgoing, balanced, moderate hub incoming, and strong hub incoming structures. Probability $p_{in} = 0.1$ created a network in which the majority of the synapses of the hub were outgoing (strong hub outgoing). A p_{in} of 0.5 rests in a "balanced" network in which the hub had an approximately equal number of outgoing and incoming synapses. Conversely, a p_{in} of 0.9 generated a network in which the majority of synapses of the hub were incoming (strong hub incoming).

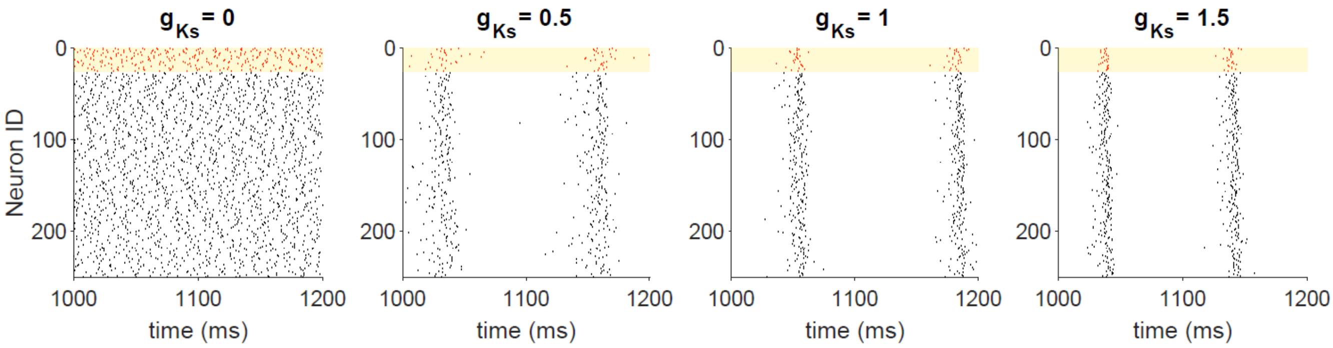

Figure S2. Examples of network dynamics under different levels of ACh modulation. Raster plots displaying 200ms of neuronal spiking activity from simulations of different g_{Ks} levels within the balanced network configuration. Neurons were sorted in descending order from the highest degree neuron to lowest. The yellow zone and red marks, respectively, denote the hub neurons and their spiking activity. The raster plots showed the temporal tightening of spiking activity between neurons as g_{Ks} was increased.

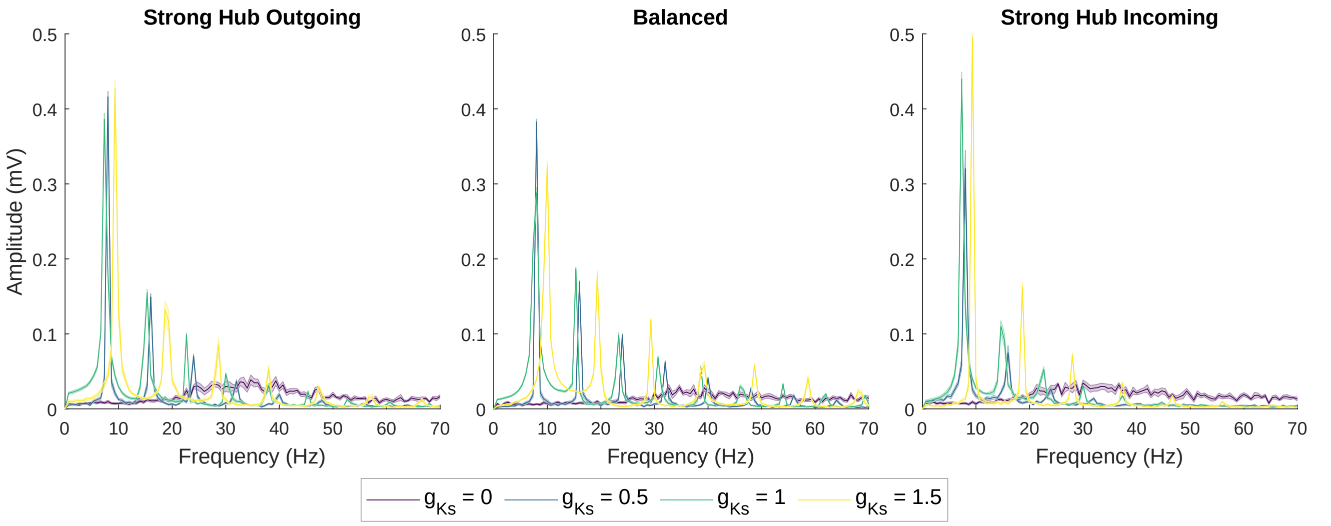

Figure S3. Spectral analysis of the local field activity (LFP) at different levels of ACh modulation, for the strong hub outgoing (left), balanced (center) and strong hub incoming (right) network configurations. All networks show incoherent activity for $g_{Ks} = 0$, with oscillations emerging for higher values of g_{Ks}.

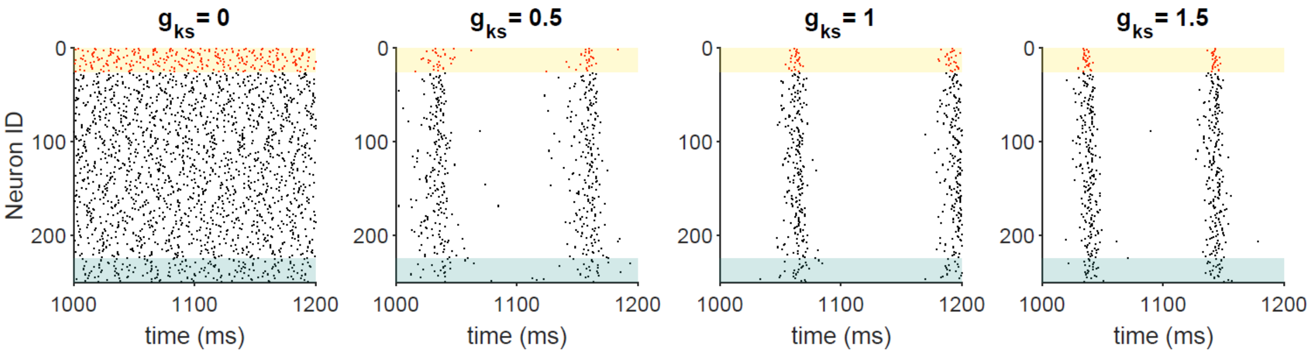

Figure S4. Dynamics of acetylcholine-modulated neuronal networks with excitatory and inhibitory neurons. E-I networks were implemented by allowing 10% of neurons to have inhibitory synaptic connections. Raster plots of 200ms segments of E-I network simulations for different levels of g_{Ks} in the balanced network configuration. Neuron IDs were sorted in a descending order from highest degree to lowest. Yellow and blue zones highlight activities corresponding to hub neurons and inhibitory neurons, respectively.

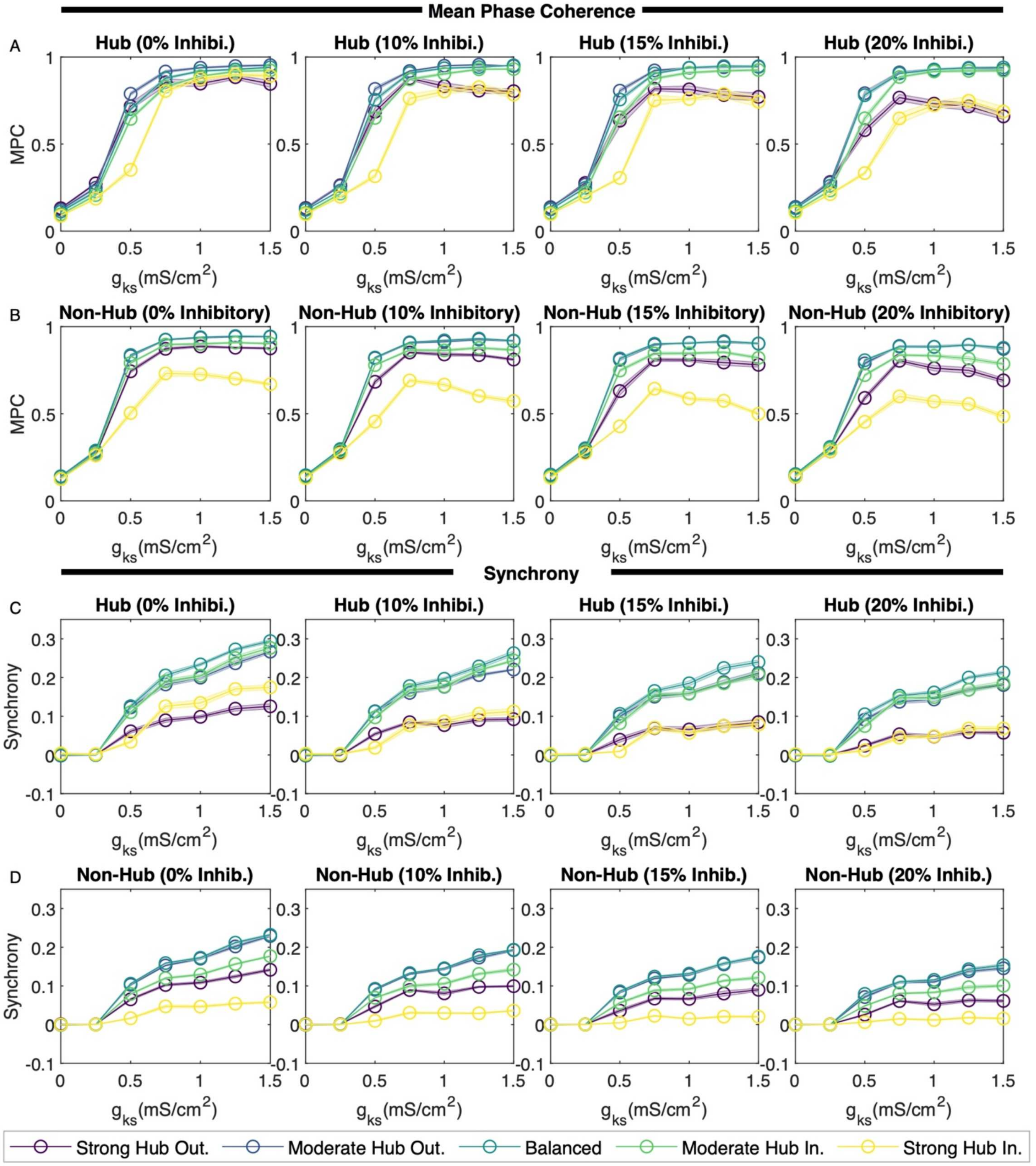

Figure S5. Comparison of group dynamics for different populations of inhibitory cells $(0\%, 10\%, 15\%, 20\%)$. We compare phase coherence and synchrony in the 4 groups for hub neurons (A, C respectively) , and non-hub neurons (B, D respectively).

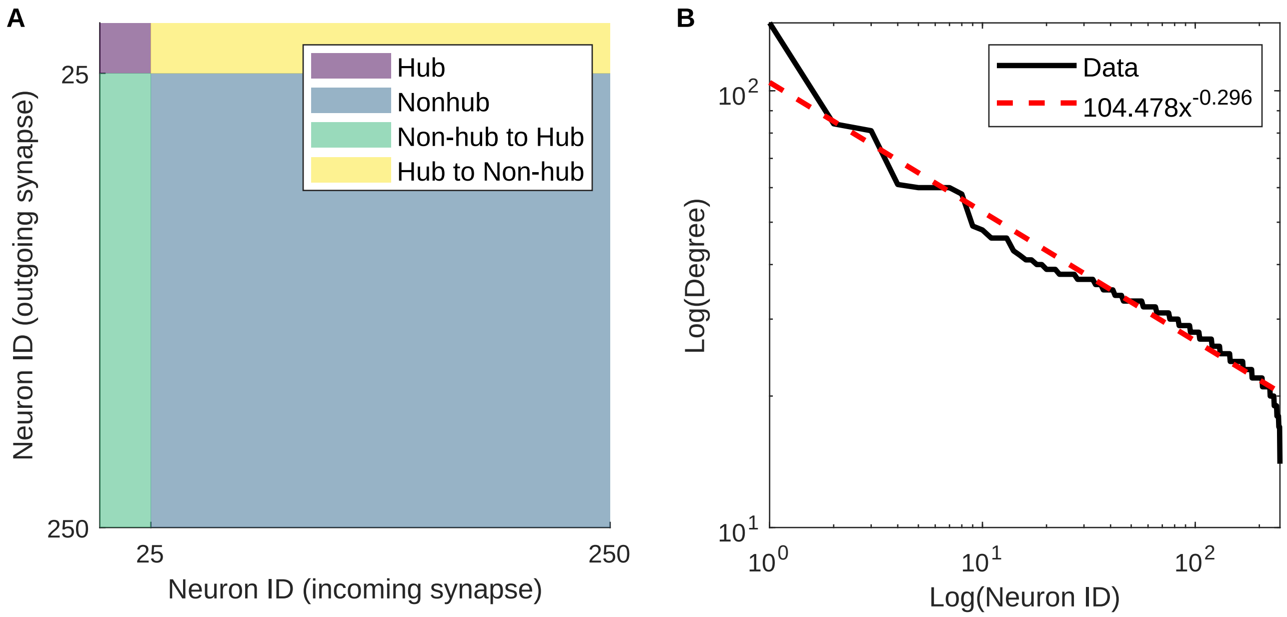

Figure S6. Zone subdivision of network connectivity. (**A**) The box represents a connectivity matrix. Neurons are aligned by degree on the x and y axes, with the neuron at label 1 having the highest degree. The x axis represents incoming synapses, while the y axis outgoing connections, such that an entry at (x_0, y_0) describes the strength of the synapse from the neuron labeled y_0 to the neuron labeled x0. Thus, the connections in the upper-lefthand corner (purple) represent connections within the hub. The yellow box represents the connections from non-hub neurons to hub neurons, and the green box the reverse. Connections between non-hub neurons are described by the blue box. (**B**) neuronal degrees; ordering from highest to lowest on a log-log scale. The power law exponent is $\alpha = -0.296$.

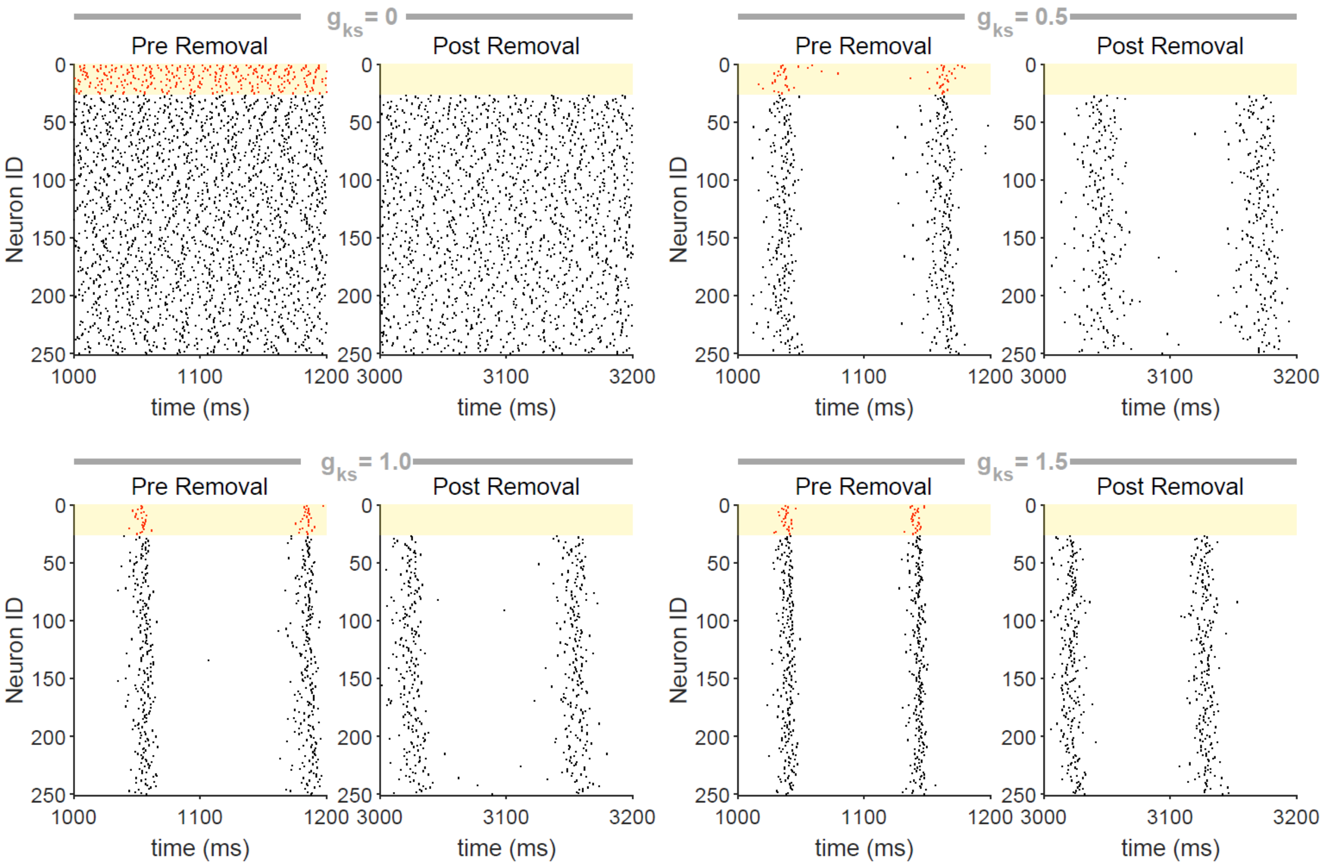

Figure S7. Effects of neuronal hub removal on ACh-modulated network dynamics. Sample raster plots showing 200ms segments of neuron firing activity pre- and post-hub ablation at for different g_{Ks} levels (yellow zones highlight the hub neurons).
